# Simulation analysis of the effect of single-chamber double-line pipe jacking through different soil materials on surface uplift and subsidence

**DOI:** 10.1371/journal.pone.0276366

**Published:** 2022-10-21

**Authors:** Xingli Jia, Zexuan Jiao, Shuaifeng Liu, Hua Zhang, Boming Shang

**Affiliations:** 1 School of Highway, Chang’an University, Xi’an, Shaanxi, China; 2 Shaanxi Construction Engineering Group Co., Ltd., Xi’an, Shaanxi, China; 3 Shaanxi Huashan Road and Bridge Group Co., Xi’an, Shaanxi, China; University of Genova, ITALY

## Abstract

This article is based on the relocation project of the 330 kV overhead line in Xi’an, China. In this paper, the soil settlement under different jacking depths was calculated by using the modified Peck’s formula. Meanwhile, by modeling in ABAQUS, the jacking process of a single-chamber double-line large diameter pipeline under different soil conditions was simulated, and the ground deformation data under the different simulated working conditions were obtained. The results of the two methods were compared with the construction monitoring results, and it was found that the finite element simulation results were closer to the actual results. The control variable method was used in the analysis of the surface soil deformation law to analyze the effect of different soil parameters and pipe jacking depths on surface soil deformation. Finally, the best soil conditions applicable to single-chamber double-line large diameter pipe jacking construction were obtained through comparative analysis. The results show that (1) when using double-line construction, the maximum surface settlement under different soil conditions is located 11–15 m from the centerline of the soil above the pipeline, the minimum settlement location is inside the isolation pile, and with the increase in jacking distance, the settlement at the same section of the surface will gradually decrease and finally produce a small uplift. (2) In the first jacking, the settlement of powder clay is the largest, and the maximum settlement points in the surface section are more distributed. The maximum settlement value is approximately 11.66 mm. The settlement of powder soil is the smallest but produces a certain uplift deformation, and its maximum settlement is more concentrated in the surface section. After the comparison of deformation and soil parameters, loess-like soil is more suitable for single-compartment double-line large diameter pipe jacking construction. (3) When the top pipe burial depth changes, the greater the burial depth is, the smaller the settlement but the greater the lateral influence range. In the soil parameters, the modulus of elasticity only changes 3 MPa, and the settlement change value is approximately 5 mm. By changing the parameters, it can be obtained that the larger the modulus of elasticity of the soil is, the smaller its deformation. The larger the internal friction angle of the soil is, the smaller its deformation, but the maximum value of settlement change is only 1.7 mm, which means that the change in the internal friction angle has little effect on the soil deformation.

## Introduction

Currently, the utilization of underground space in municipal engineering has received increasing attention. As a trenchless technology for underground passages, pipe jacking has been widely used in underground engineering construction. However, there are many factors to be considered in its construction, such as adverse effects on surrounding roads, buildings, residential areas, etc., especially the construction of large diameter pipelines, and the spatial overlap of pipelines should also be considered. Its construction process will disturb the surrounding soil, which will lead to a certain deformation of the ground surface. This deformation, if too large, can cause harm to roads and buildings [1].

In addition, different soil materials show different deformation traits during pipe jacking construction, and the applicability of pipe jacking construction urgently needs to be clarified. At present, in terms of the analysis methods of the surface deformation of pipe jacking construction, there exist mainly numerical simulations to establish the empirical formula, software modeling for construction simulation, and collection of deformation data. From the aspect of deformation data statistics, Namin et al. [2] illustrated that the construction of integrated pipe corridors under top pipe construction has far more advantages than disadvantages on the surrounding environment through statistical measurement data elaboration. Robati and Atabi [3] used SPASS to collect and analyze large data to illustrate the impact of pipe corridors on the urban environment during the construction process and the operation phase after the completion of the corridor. In terms of software simulation, Zhang [4] used the geotechnical finite element software Midas GTS NX to numerically simulate rainfall on the slope of an underground integrated pipe corridor pit and the contact between the pipe corridor structure and the soil. Liu [5] used Midas GTS software to establish a 3D numerical analysis model to analyze the deformation of the support structure at each construction stage, i.e., the support structure with the jacking distance. The force of the support structure becomes parabolic as the jacking distance increases. In terms of extracting data from dependent projects and conducting simulations, Tang [6] studied the surface settlement pattern and soil deformation prediction caused by shield excavation tunnels and the characteristics of fissure-containing end-plus solids using the Shenyang underground integrated pipe corridor as the engineering background. Lin and Gong [7] studied the surface settlement caused by the construction of straight and curved tunnels using the shield method and jacking method using a shield tunnel as the engineering background. The surface settlement curves obtained by the two methods were analyzed by numerical simulation. Compared with that of the linear tunnel, the surface settlement trough of the curved tunnel shifted approximately 0.5 d in the curved direction, and the total difference between the surface settlement of the shield method and the pipe jacking method of the linear tunnel was smaller, while the surface settlement of the curved tunnel by the pipe jacking method was larger than that of the shield method. Time and cost can be saved when using pipe jacking construction in special conditions instead of setting up anti-slip dams for excavation construction. In addition, the deformation results were calculated using empirical formulas first, and then construction simulations were conducted with finite element software. Finally, the results obtained from both were compared with the monitoring data to obtain the maximum value of soil settlement above the pipe axis [8–12]. In terms of the factors affecting the ground uplift, including the additional stress or friction, the corresponding soil parameters, the construction method under special conditions, and the water pressure of the pipe, Niu et al. [13] analyzed the ground deformation caused by additional stresses and excavation surface friction during pipe jacking construction by varying the jacking depth of pipe jacking. Yen and Shou [14] used model connected finite element analysis and the displacement control method to estimate the jacking force required for pipe jacking construction and improved the model’s jacking force for comparison. The results obtained were compared with monitoring data to verify the applicability of the model, and the results showed that the model can estimate the jacking force in the middle and late stages of the pipe jacking process more accurately. Xia [15] considered that the groundwater level changes with the change in the surrounding environment and obtained the stresses, settlement deformation of different parts of the integrated pipe corridor and the change law of soil stress around the corridor by numerical simulation for a large integrated pipe corridor with a determined burial depth. Maehara et al. [16] injected fatty acids as lubricant between the pipe and the surrounding soil to reduce the frictional resistance. With the increase in fatty acid addition, the leakage rate of the filler decreased, but excessive addition would lead to water absorption in the pipe, and the addition amount of approximately 3% was the best for soil settlement reduction. Considering the interaction between the fluid, pipe and soil when performing jacking construction of a loaded water pipeline, the displacement and stress of the buried pressurized water pipeline installed by the jacking method should also be studied, and the displacement of the concrete pipe joints and the stress are significant under unsteady flow conditions, in addition to the maximum tensile stress exceeding the tensile strength of the concrete due to the pressure transient effect, leading to the generation of cracks and leakage [17–20]. In addition, the pipe jacking method can be used in completed subway passages, underground mines and other measures to precisely control the horizontal and vertical displacements through numerical simulation and field monitoring. The study shows that when accurate simulation is used to control the displacement, the vertical displacement of the tunnel mainly goes through three different stages: the initial settlement stage, rapid uplift stage and stable uplift stage [21–23]. For surface settlement caused by underground construction, some researchers have also simulated the construction of shield projects by modeling with finite element software to analyze different working conditions or soil parameters and conditions to obtain the surface settlement data [24–26]. Most of the univariate research methods for specific materials use the method of controlling variables. There are many studies that control a variable through experimental calculation or software simulation to obtain the effect [27, 28].

Single-line pipe jacking construction is restricted by underground conditions such as buildings and soil layers. The use of a single-cabin double-line passage will greatly reduce the workload, but there are few studies on the surface subsidence deformation caused by this method. It is of great significance to explore the surface soil subsidence of double-line jacking. This paper uses finite element software modeling for construction simulation and compares it with monitoring data, adjusts soil parameters under different conditions, and simulates surface settlement data during construction. Finally, the influence of different soil conditions on the surface deformation is simulated, and suitable construction conditions for single-chamber double-line large diameter pipe jacking are obtained.

## Materials

### Soil layer parameters

The method of pipe jacking in this project adopts the soil pressure balance method, which uses the pressure in the soil bin and the discharge of the conveyor to balance the groundwater pressure and soil pressure. After one section of the pipe is jacked into the soil, the second section of the pipe is lowered to continue jacking. The principle is that with the help of the main jacking cylinder and the thrust between pipes and relays, the tool pipe or roadheader is pushed from the working pit through the soil layer until it is lifted in the receiving pit, and the pipe is buried between the two pits immediately after the tool pipe or roadheader [29]. To simulate pipe jacking construction under different soil conditions, the soil parameters for each stratigraphic condition at the site need to be obtained. Based on the stratigraphic information from the site exploration, the foundation soils within the depth range of 25 m below the natural ground level of the proposed site, from new to old for each stratigraphic structure, are divided as follows.

Miscellaneous fill (${\text{Q}}_{4}^{\text{ml}}$): The composition is complex. The layer bottom burial depth is 0.30~2.80 m, and the layer bottom elevation is 366.60~379.03 m.
Plain fill soil (${\text{Q}}_{4}^{\text{ml}}$): It is mainly powdered clay. The depth of the layer bottom is 0.50~3.00 m, and the elevation of the layer bottom is 365.36~379.48 m.
Loess-like soil (${\text{Q}}_{4}^{\text{al}}$): The depth of the layer bottom is 1.50~10.00 m, and the elevation of the layer bottom is 363.19~372.72 m.
Powdery clay (${\text{Q}}_{4}^{\text{al}}$): There is a small amount of clay. The bottom of this layer is buried at 5.00~23.00 m, and the bottom elevation of the layer is 347.75~366.59 m.
Powder soil (${\text{Q}}_{4}^{\text{al}}$): It is dominated by clay. The depth of the bottom of the layer is 1.10~16.00 m, and the elevation of the bottom of the layer is 362.66~367.70 m.
Coarse sand (${\text{Q}}_{4}^{\text{al}}$): It is mainly composed of quartz and feldspar. The depth of the bottom of the layer is 9.8~17.0 m, and the elevation of the bottom of the layer is 358.49~368.20 m.
Gravelly sand (${\text{Q}}_{4}^{\text{al}}$): It is mainly composed of quartz and feldspar. The depth of the bottom of the layer is 11.7~15.5 m, and the elevation of the bottom of the layer is 362.36~364.48 m.

According to the depth of pipe jacking construction and several common soil layer types, 4 common soil layers that this project crosses are selected for pipe jacking construction simulation [29], i.e., loess-like soil, silty soil, powdery clay and clay. The soil parameters of the 4 soil layers are shown in Table 1.

**Table 1:** Soil parameters of the soil layers.

Soil layer	Natural heavy gamma (kN/m^3^)	Modulus of elasticity E (MPa)	Poisson’s ratio υ	Friction angle φ (°)	Cohesion c (kPa)
Loess-like soil	20.0	20.0	0.27	30	35.0
Powdered earth	20.5	25.0	0.26	30	40.0
Powdery clay	20.6	10.8	0.31	22	38.0
Clay	20.4	19.1	0.35	15	55.0

### Relying project

**Fig 1:**
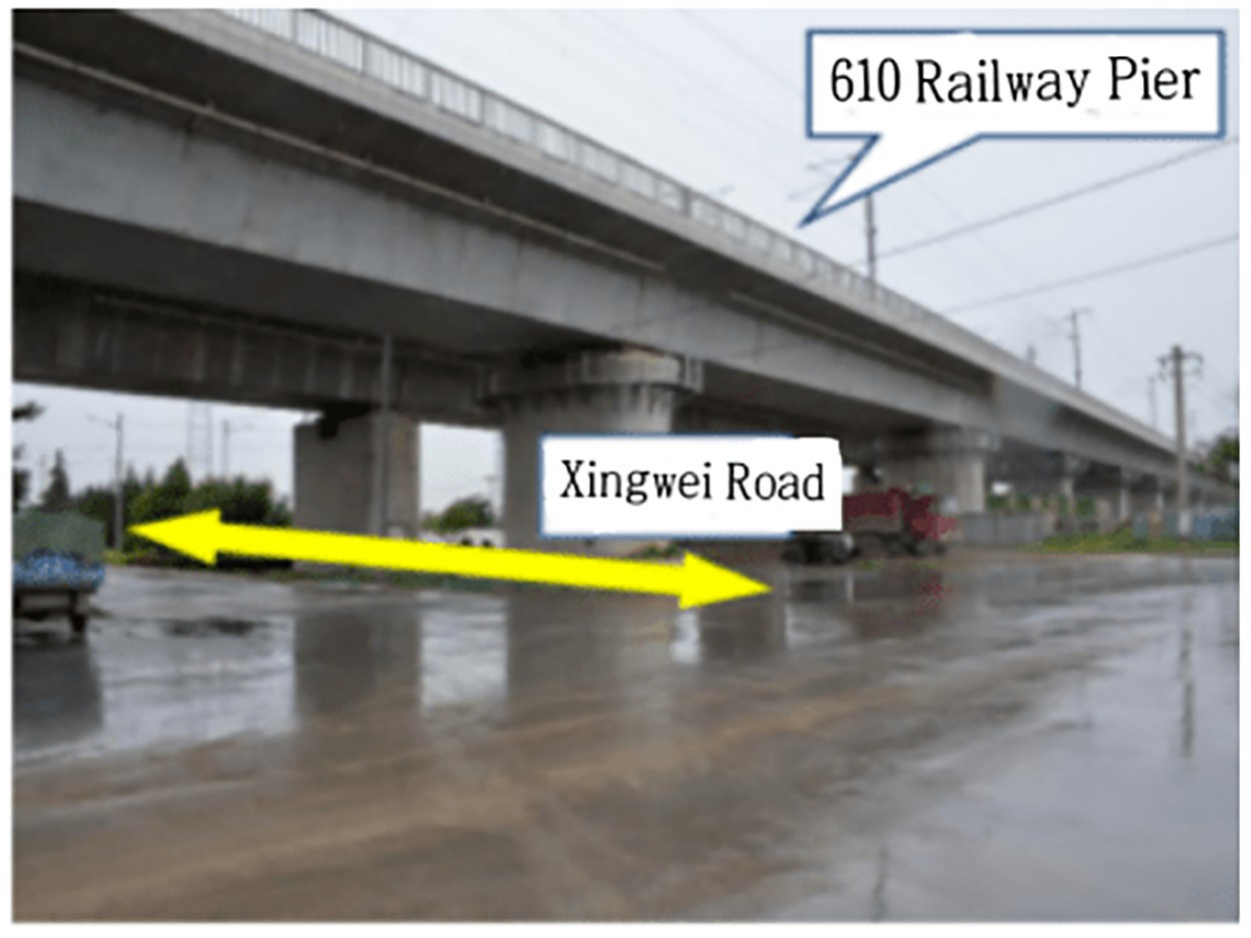
Overview of the construction section.

To obtain more accurate simulated soil parameters and at the same time make the pipe jacking information closer to reality, the simulation will rely on the 330 kV overhead cable relocation project in Xi’an, China. This project passes through the Zhengxi High-speed Railway Bridge. To avoid bridge piers at approximately 100 m away from the high-speed rail piers, the square pipe is divided into a single cabin and double lines with the pier as the centerline to continue excavation. The angle between the two lines is 36°. The horizontal distance between the two wells is 15 m, and the jacking form is a separate single chamber of 2×Φ3.5 m. The mud-water balance jacking method is adopted to transport waste soil with mud water as the medium. The length is approximately 0.22 km, the burial depth of the starting well is approximately 12 m, and the burial depth of the ending well is approximately 20 m. The construction section overview is shown in Fig 1.

The pipe jacking method tunnel uses prefabricated reinforced concrete pipe sections with an inner diameter of 3500 mm, a wall thickness of 320 mm, an outer diameter of 4140 mm and a single section length of 2.5 m. The pipe sections are made of C50 concrete with a seepage resistance grade of P10, and steel socket connections are used between the pipe sections. The average burial depth of the pipe is 8.5 m.

**Fig 2:**
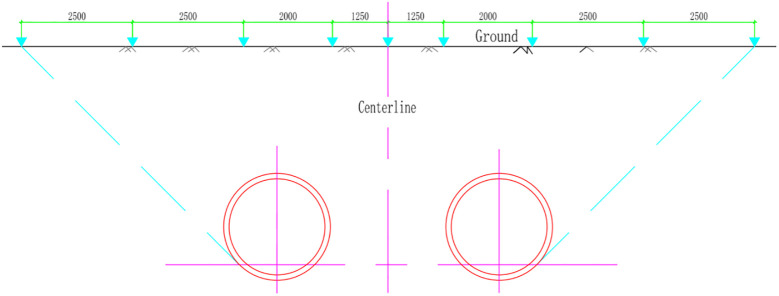
Cross-sectional monitoring point arrangement.

In addition, the project will monitor the vertical displacement of the ground surface in real time during the construction process, that is, the cumulative vertical change and the change since the last time. The horizontal distance between the vertical displacement monitoring points is not more than 20 m. The ground vertical displacement monitoring section starts from the centerline of the two pipelines, with 4 monitoring points on the left and right. The interval between the monitoring points is 5–10 m. When the distance between the measuring points is less than 2 m, the measurement points are combined. The layout of the section monitoring is shown in Fig 2.

The data of the 24th time after the pipe jacking started, which was approximately 5 m from the excavation section, were selected as a reference, and the data are shown in Table 2.

**Table 2:** Dependent project monitoring data.

Observation point number	Elevation (m)	Change amount (mm)	
		This time	Grand total
CJ217	400.12811	-0.24	-4.58
CJ218	400.18182	-0.22	-7.46
CJ219	400.12451	-0.22	-2.89
CJ220	400.15081	-0.29	-2.49
CJ221	400.16910	-0.22	-2.49
CJ222	400.12646	-0.21	-2.88
CJ223	400.18773	-0.24	-7.78
CJ224	400.15479	-0.23	-4.50

## Methods

### Numerical calculation

Currently, ground settlement caused by pipe jacking construction is usually predicted using the ground settlement trough theory proposed by Peck, which assumes that construction-induced ground settlement occurs without drainage, so the volume of the surface settlement trough should be equal to the volume of ground loss, and the lateral distribution of ground settlement is similar to a normal distribution curve, calculated as follows:

$${\text{S}}_{\left(\text{x}\right)}={\text{S}}_{\text{max}}\;\text{exp}\left(-\frac{{\text{x}}^{2}}{2{\text{i}}^{2}}\right)$$ (1)

$${\text{S}}_{\text{max}}=\frac{{\text{V}}_{\text{s}}}{\sqrt{2\pi }\text{i}}$$ (2)

$$\text{i}=\frac{\text{Z}}{\sqrt{2\pi }\text{tan}\left(45{}^{\circ}-\varphi /2\right)}$$ (3)

$${\text{V}}_{\text{s}}=\eta \cdot \text{A}$$ (4)

S_(x)_ is the ground settlement at calculation point x; x is the lateral horizontal distance from the calculation point to the axis of the jacking pipe; S_max_ is the maximum ground settlement above the axis of the jacking pipe; i is the lateral horizontal distance from the countercurve point of the settlement tank curve to the axis of the jacking pipe; φ is the angle of internal friction of the soil; Z is the thickness of the overburden from the center of the pipe to the ground; V_s_ is the amount of soil loss at the excavation surface; η is the rate of soil loss; and A is the area of the excavation surface of the pipe.

The abovementioned soil loss rate is the key, which indicates the amount of soil loss per unit length, mainly due to 8 reasons, such as the pipe jacking machine and pipe annular gap, construction overexcavation control, interrelief sealing, pipe wall friction, pipe correction, back soil deformation, pipe jacking in and out of the hole, pipe section rebound, etc. Drawing on the experience of the shield, the soil loss rate decreases with the growth of the pipeline burial depth, and its value is concentrated in the range of 0.20%-2.0%. The selected value of the soil erosion rate can be based on the experience of the shield structure. By analyzing the existing studies on shield jacking, it is known that the erosion rate decreases with increasing pipeline burial depth, and most shield construction erosion rates are 0.20%-2.0%. According to the actual data of the supported project, we can calculate that the sink inflection point i = 5.873 m for single-line pipe jacking and the maximum ground settlement S_max_ above the pipe jacking axis = 3.65 mm. S_max_ The maximum curvature point of the curve is used as the lateral settlement influence range of pipe jacking, and the influence range of pipe jacking is the center axis of the pipe to each side = 10 m. $\text{B}=\sqrt{3}\text{i}$. The lateral distribution curve of soil settlement for single-sided pipe jacking is as follows:

$$S_{\left(\text{x}\right)}=-0.0365\text{exp}(-0.0145{\text{x}}^{2})\;\left(\text{unit}:\text{m}\right)$$ (5)

**Fig 3:**
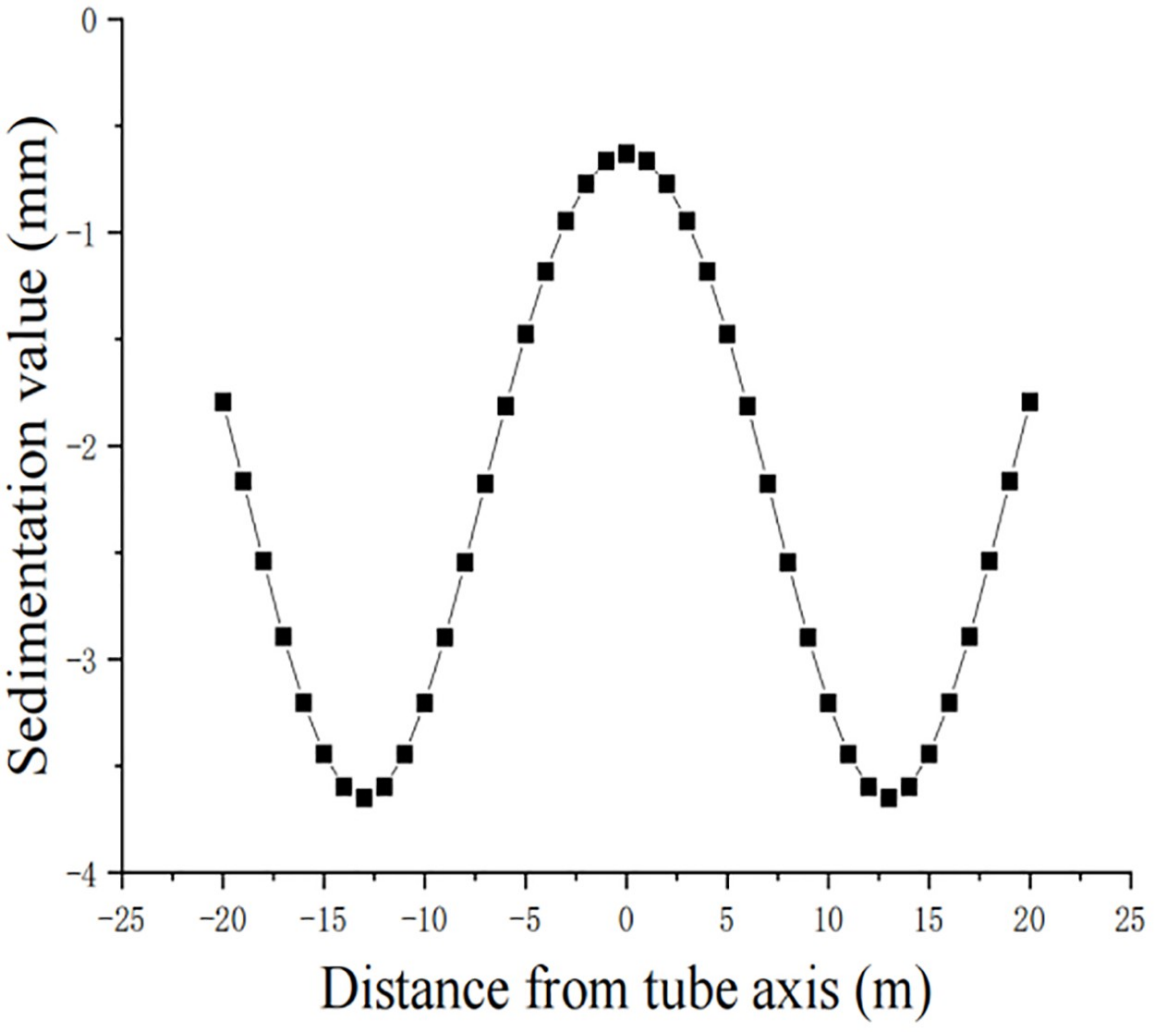
Peck’s formula for simulating a bilinear settlement curve.

In this project, the construction method of single-compartment double-line simultaneous jacking is adopted, and the working conditions of the two compartments are basically the same. Without considering the buildings between the two lines, the ground settlement curve of the double line can be regarded as two single-line settlement curves separated by a certain distance, so the neutral axis of the double line can be superimposed on the single-line formula for correction, and the lateral settlement curve of double-line pipe jacking is obtained as follows:

$${\text{S}}_{\text{d}\left(\text{x}\right)}=0.0365\{\text{exp}\left[-0.0145{\left(\text{x}+{\text{x}}_{0}\right)}^{2}\right]+\text{exp}\left[-0.0145{\left(\text{x}-{\text{x}}_{0}\right)}^{2}\right]\}$$ (6)

where S_d(x)_ is the amount of ground settlement (in m) at x from the center of the double line caused by double-line pipe jacking. x_0_ is the distance from the center of the single pipe to the center of the double line. The other symbols are the same as in the single-line settlement formula. According to the dependent engineering conditions, the settlement curve of the double line is shown in Fig 3.

According to the analysis in Fig 3, the settlement curve of the two lines obtained by Peck’s formula is similar to two normally distributed curves, and the settlement values and trends of the two pipelines are the same because the existence of the intermediate buildings and the distance between the two pipelines are not considered. The maximum settlement value is located above the pipeline, and the numerical simulation shows that the maximum settlement value is 3.6 mm, which is consistent with the results obtained from the above single-line calculation. The settlement decreases gradually from the middle axis of the pipeline to both sides, and the soil in the middle produces a bulge.

**Fig 4:**
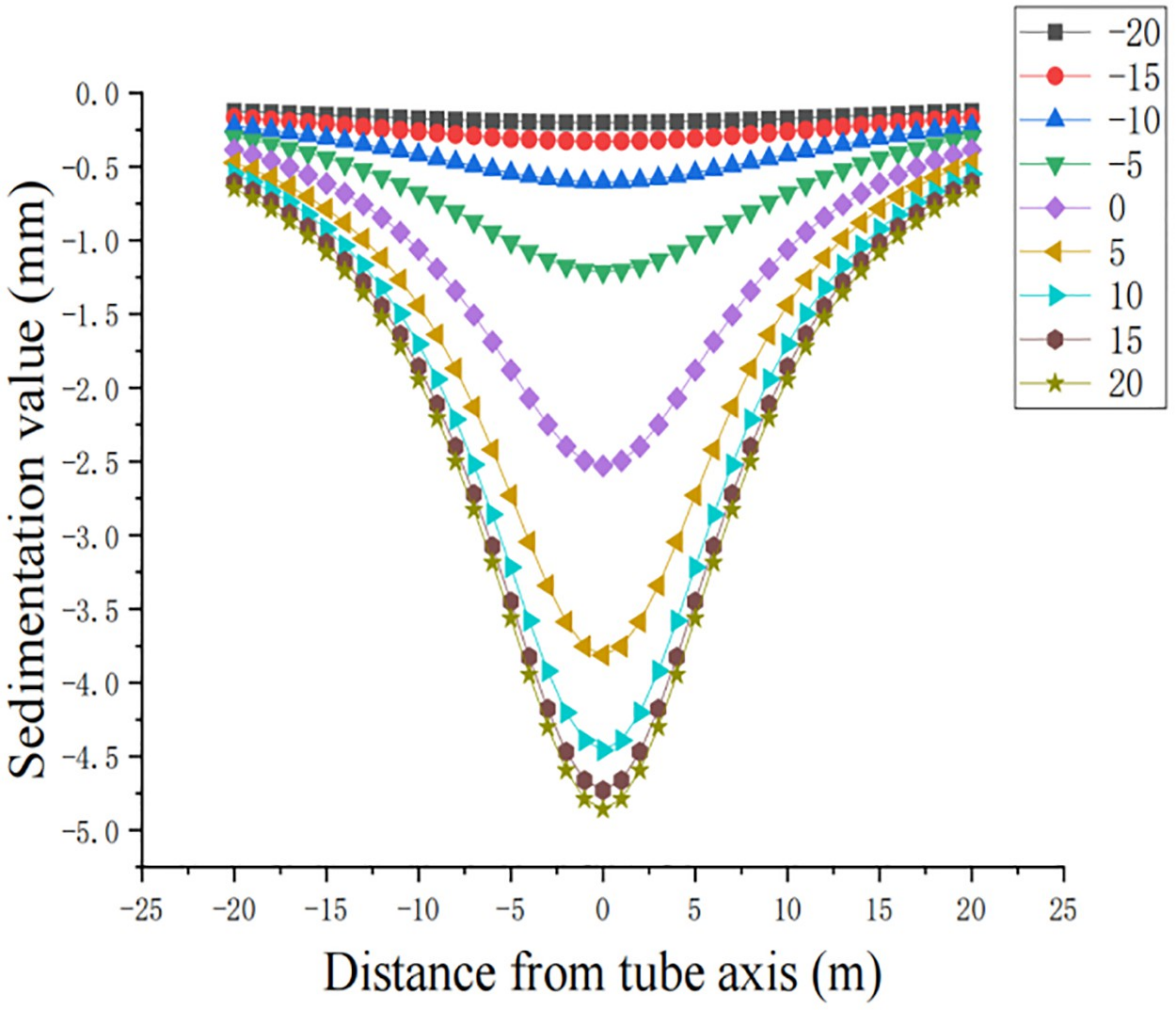
Soil settlement curve from the excavation surface to different locations.

The maximum settlement S_max_ after considering Poisson’s ratio of soil is obtained according to the dependent engineering calculation. The soil settlement curves from the excavation surface to different locations are shown in Fig 4.

According to the analysis of Fig 4, it can be seen that the excavation surface starts to settle at 20 m in front of the monitoring section, and as the excavation surface advances forward, the excavation surface continues to settle with a settlement value of less than 1 mm. The excavation surface is 5 m in front of the monitoring section and 15 m after passing, and the stratum has a large settlement. It can be seen through the figure that the lateral influence range of the single-side jacking pipe is approximately 25 m.

### Finite element simulation

The theoretical basis of the finite element method is the theory of minimum potential energy, which means that when the potential energy of a system is minimal, the system will be in stable equilibrium. When using the finite element method, the structure to be analyzed is first meshed and then solved with the help of a suitable solution method to calculate the displacements and strains occurring at the cell nodes, and the structural stresses are calculated. The main ideas and solution steps are structural discretization, cell characterization, cell set analysis, and introduction of boundary conditions to solve for unknown node displacements. The software chosen is ABAQUS, a large engineering simulation software based on the discrete idea of finite elements, and it has a rich unit model, material model and analysis process, which has strong applicability in the field of geotechnical engineering. In this paper, based on the 330 kV overhead cable relocation project in Xi’an, China, the finite element method is used to model, simulate the construction, and analyze the data of the project in ABAQUS software. Most underground projects involve semi-infinite or infinite domains, and finite elements usually deal with such problems by discretizing in a finite area. To reduce the error, it is necessary to ensure a sufficient range of discretization areas and to ensure that the boundary conditions outside the area are as close as possible to the actual working conditions.

**Fig 5:**
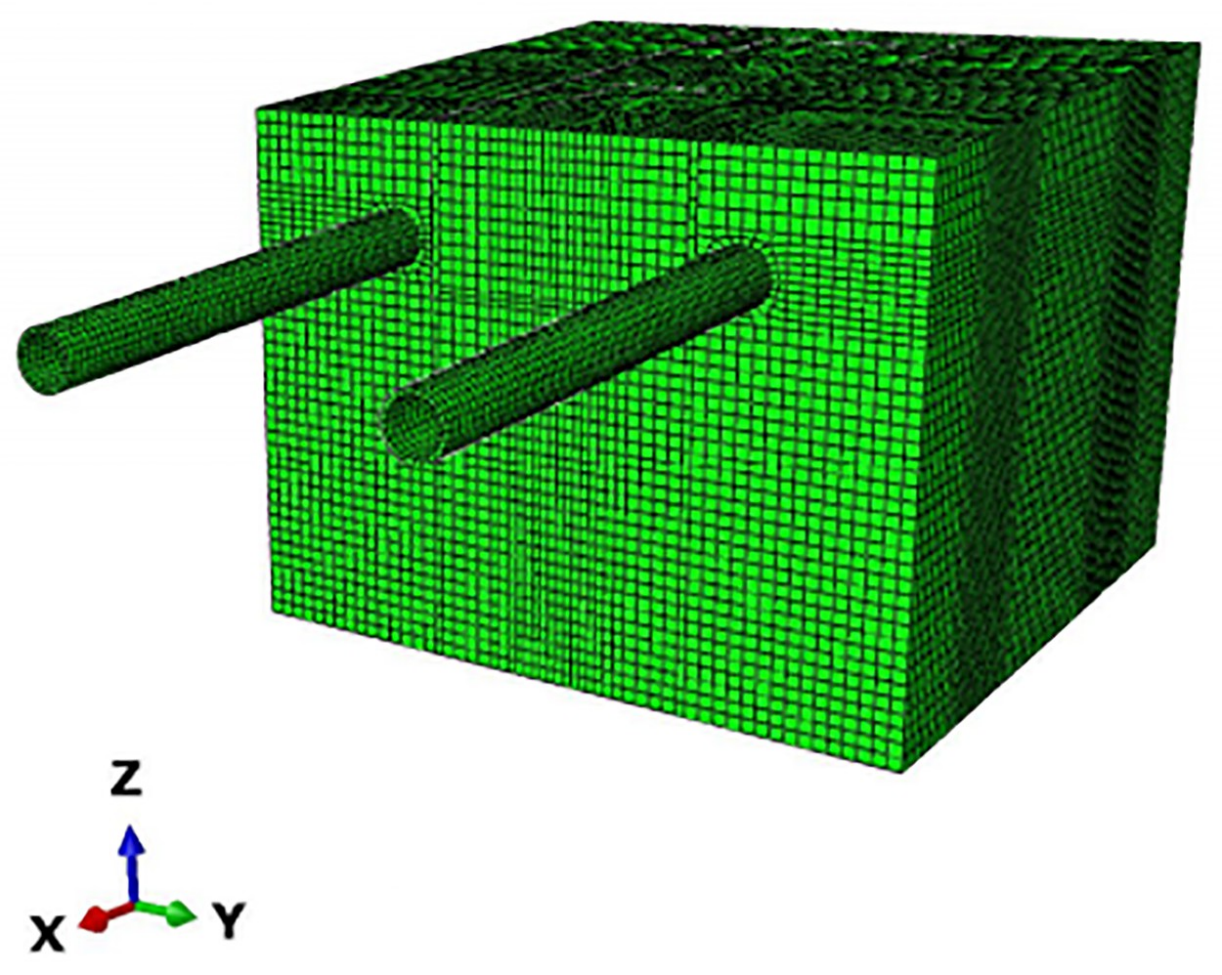
Overall model meshing results.

**Fig 6:**
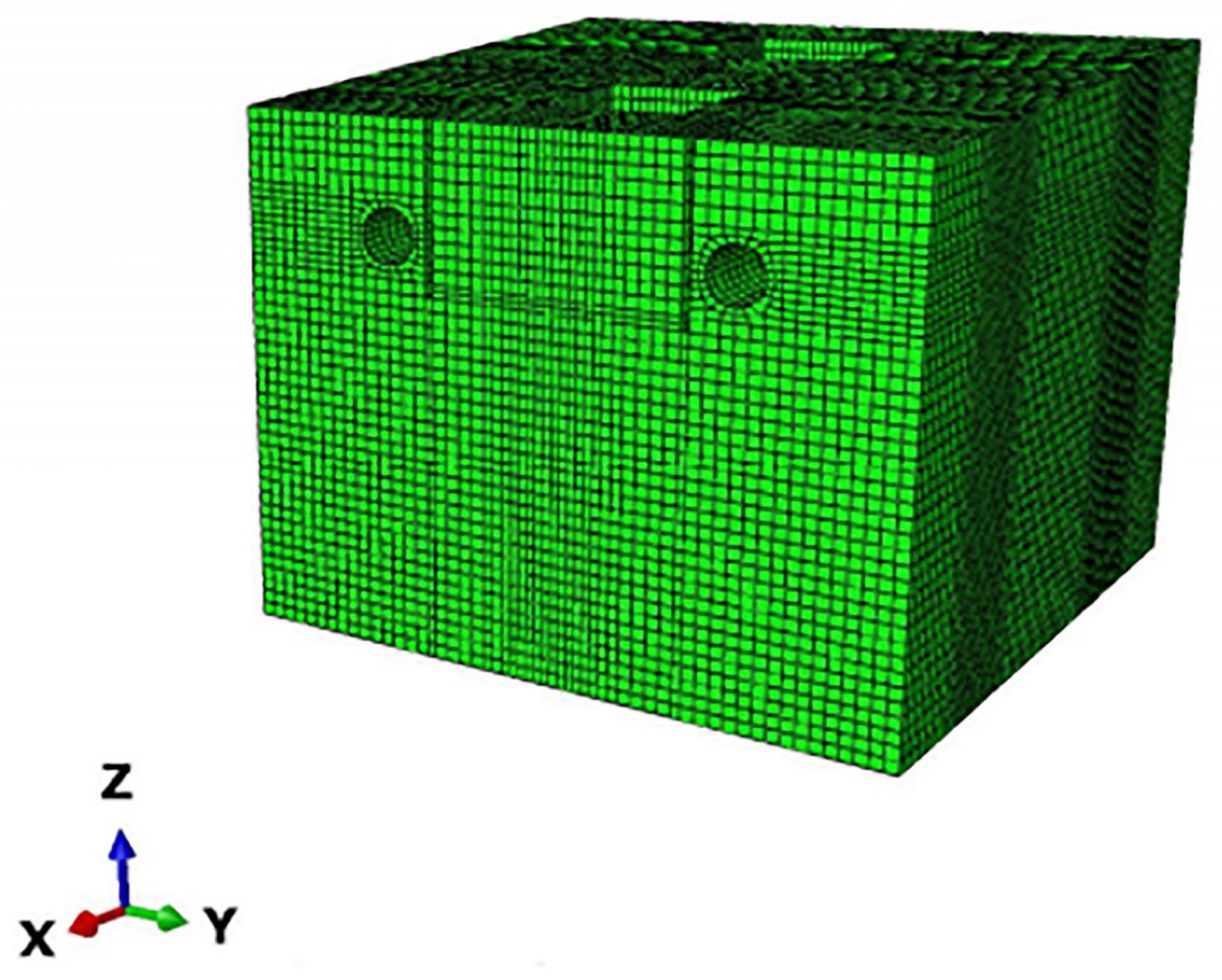
Soil model meshing results.

**Fig 7:**
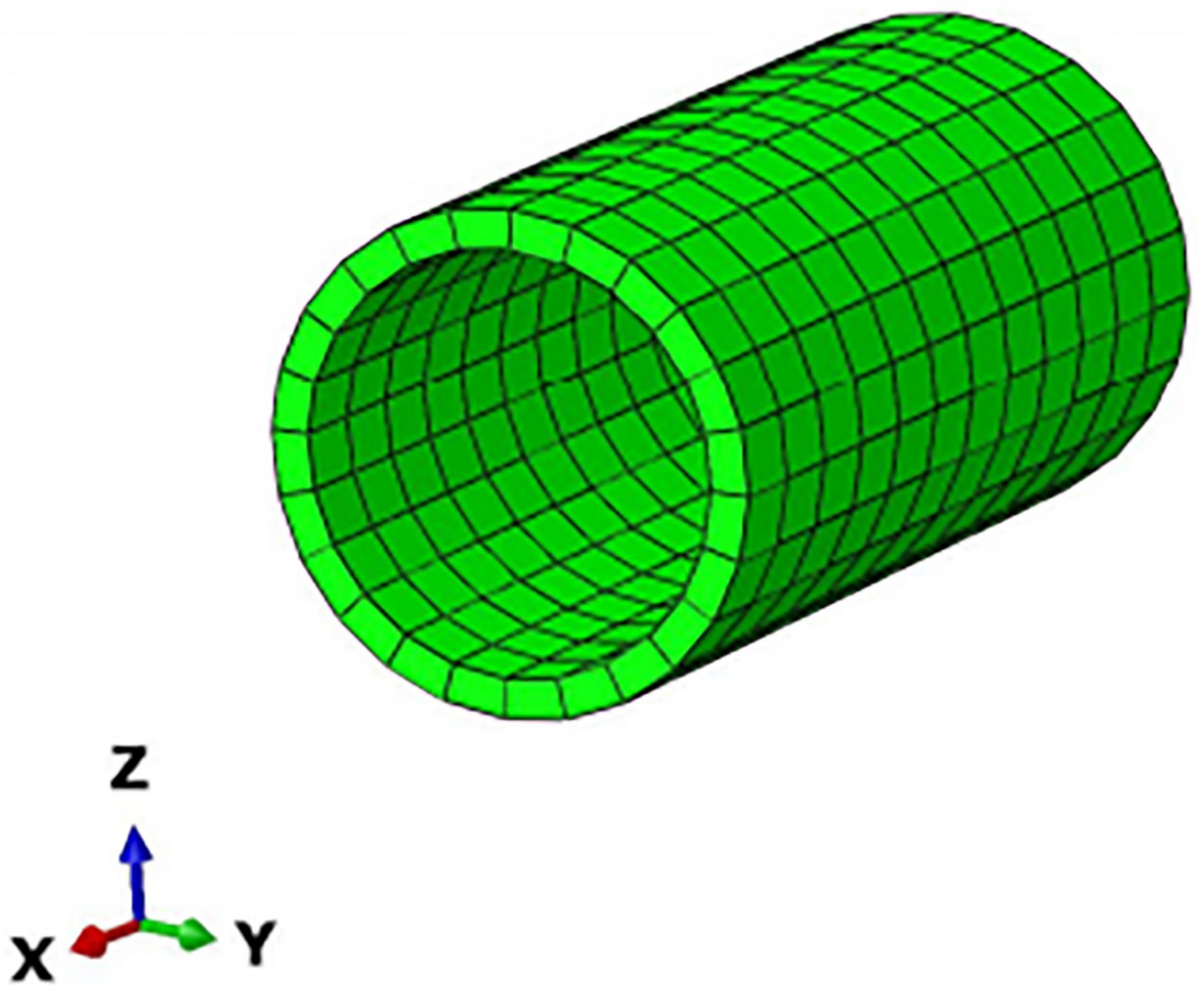
Pipe model meshing results.

From previous experience and research, it can be concluded that the stress and displacement changes caused by stress release are less than 5% outside 3 times the hole diameter and less than 1% outside 5 times the hole diameter. The surface deformation and stress effects caused by pipe jacking construction are in the range of 3 to 5 times the hole diameter, so the study of surface deformation is usually chosen to be centered on the central axis of the pipe outward to the area of 3 to 5 times the hole diameter (Mingming Cao, 2016). Because the pipe jacking section of this project is a double line at the same time and there are two piers in the middle, considering its special characteristics and the difference from single-line pipe jacking construction, the model size is established as 50 (X) × 40 (Y) × 50 (Z). The boundary conditions of the model are as follows: the left and right boundaries limit the displacement in the X direction, the lower boundary limits the displacement in the Y direction, the front and rear boundaries limit the displacement in the Z direction, and the ground is a free surface. In addition to the soil and pipeline, the model also has two high-speed railway pier bearings between the left and right lines and two 46 m long isolation piles located between the pipeline and the piers for controlling the deformation of the piers. According to the length of the pipe section, model calculations and site monitoring cross-section arrangement and other comprehensive considerations, the advance length is a total of 20 m. Because the length of the pipe section for each section is 2.5 m to reduce the calculation, a single advance length of 5 m is divided four times to complete the jacking, and the results take 5 m, 10 m, and 20 m of the surface deformation data. To show the accuracy of the model as much as possible without excessive calculation, the soil is divided into 145,560 meshes, all of which are hexahedrons. The overall model, soil and pipe meshing is shown in Figs 5–7.

In the finite element simulation of pipe jacking construction for model analysis and calculation, the soil is a continuous homogeneous isotropic ideal elastoplastic body, and the calculation takes the Moore Coulomb yield criterion, which is a yield theory that takes into account the maximum shear stress or a single shear stress acting on the positive or mean stress and is a common criterion for soil calculations. The parameters are selected using the reference values of soil parameters from the site survey. The settlement resulting from consolidation and reconsolidation of the soil after excavation is not considered. When building the pipe model, the pipe jacking head model is also built, and the influence of the head during jacking is not considered because the stiffness of the head is much larger than that of the soil body. Meanwhile, to ensure that the pipe joint material is an isotropic linear elastomer, the influence of flexible joints between pipe joints is ignored. The capacity of the pipe piece is selected as 27 kN/m^3^, and the modulus of elasticity is taken as 30,000 MPa.

When performing construction simulation, the overall model is first created, and material and interface properties are set. The soil to be removed and the pipe to be jacked are set up accordingly. The method of activating the living and dead units is used for jacking, i.e., the pipe is first put in a "dead" state, and the ground stress is balanced. Then, the pipe is placed, and the stress on the soil is released, "activating" the soil and pipe to be removed in the first step of jacking and applying gravity at the same time, thus removing the soil body from the stress. Finally, the above steps are repeated, and step-by-step jacking is completed.

### Method contrast

**Fig 8:**
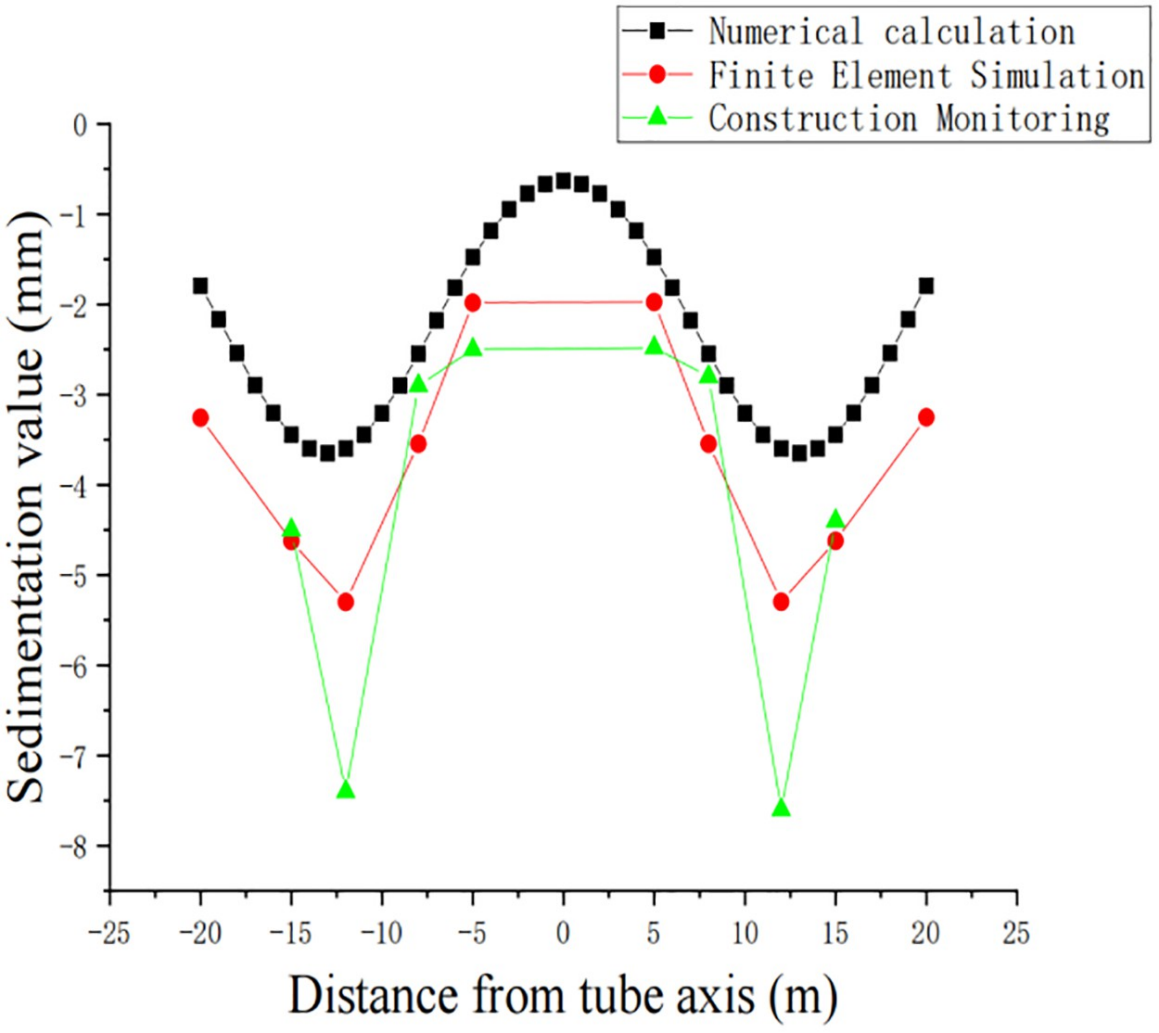
Comparison of the settlement curves of the three methods.

To obtain the surface deformation law, the numerical simulation, finite element simulation and construction monitoring results were compared. To make the finite element simulation results close to the construction monitoring results, ten points in the model with the same location as the monitoring were selected for data extraction. In the numerical simulation, the settlement curve of the double pipe simulated by Peck’s formula was used, the surface vertical settlement curve was selected for the monitoring results, and the surface settlement value of the surface section above the jacking pipe after the start of jacking was used for the finite element simulation. The results obtained from the comparison of the three methods are shown in Fig 8.

According to the analysis in Fig 8, it can be seen that for all three simulation methods, the deformation curves are similar to two normal distributions, and the trend of the surface settlement curve obtained from the on-site construction monitoring coincides with the results of numerical simulation. The results obtained from numerical simulation and finite element simulation are smaller than those from on-site monitoring. The difference between the settlement curve obtained by Peck’s formula and the maximum settlement value monitored on site is approximately 0.4 mm, while the difference between the settlement curve obtained by the finite element simulation method and the curve obtained from site monitoring is relatively small. Therefore, the finite element simulation method was chosen to further simulate the surface settlement pattern by comparing the workload and simulation realism.

## Results and discussion

### Construction simulation of the soil layer

**Fig 9:**
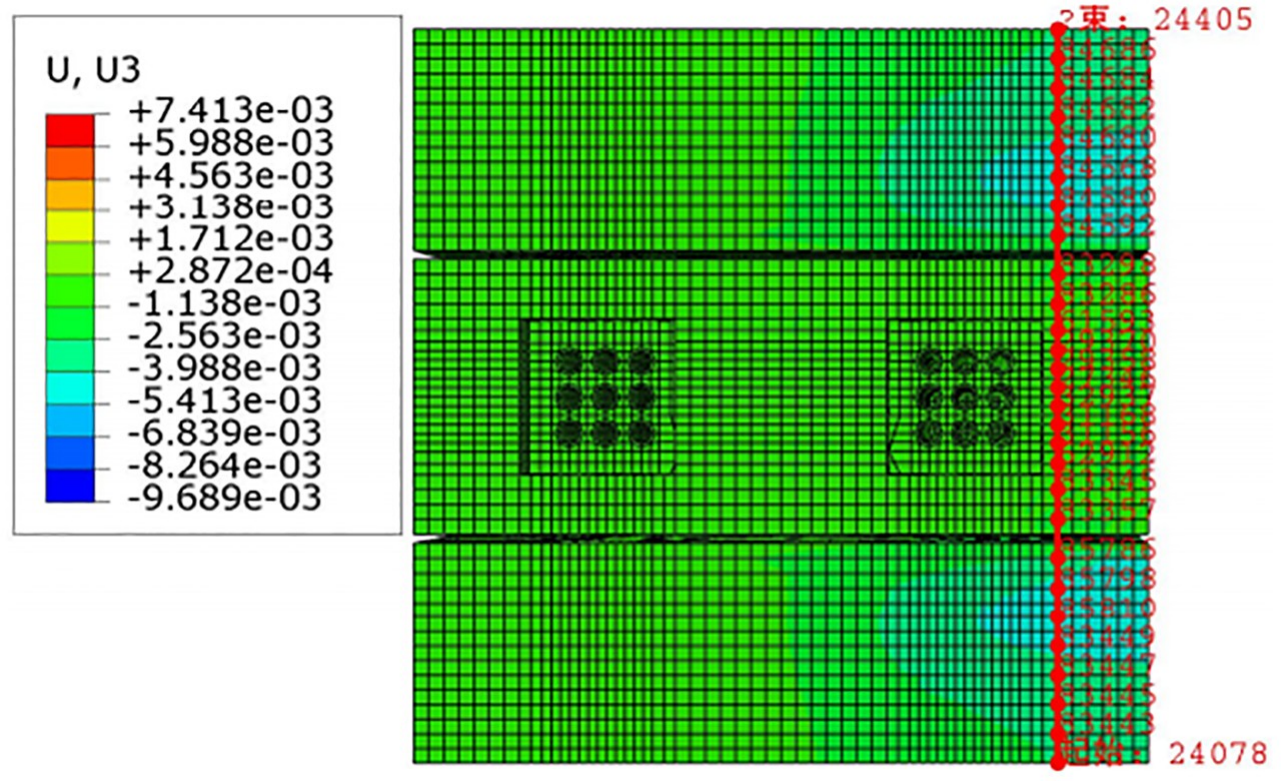
Node selection position.

After the finite element model was established, three working conditions were analyzed for pipe jacking at 5 m, 10 m and 20 m. ABAQUS writes the calculation results into an OBD format file, and the obtained conditions are independent of each other, which not only reduces the calculation volume but also facilitates the result extraction. In the selection of the surface settlement results, the surface was selected as the A-A section for the path extraction. Due to the special characteristics of this construction condition, which is a two-lane construction with a bridge pier in the middle, 14 nodes were taken on each side of the surface section with the soil centerline as the reference to ensure the accuracy of the extraction results. The nodes selected for the surface section extraction results are shown in Fig 9.

**Fig 10:**
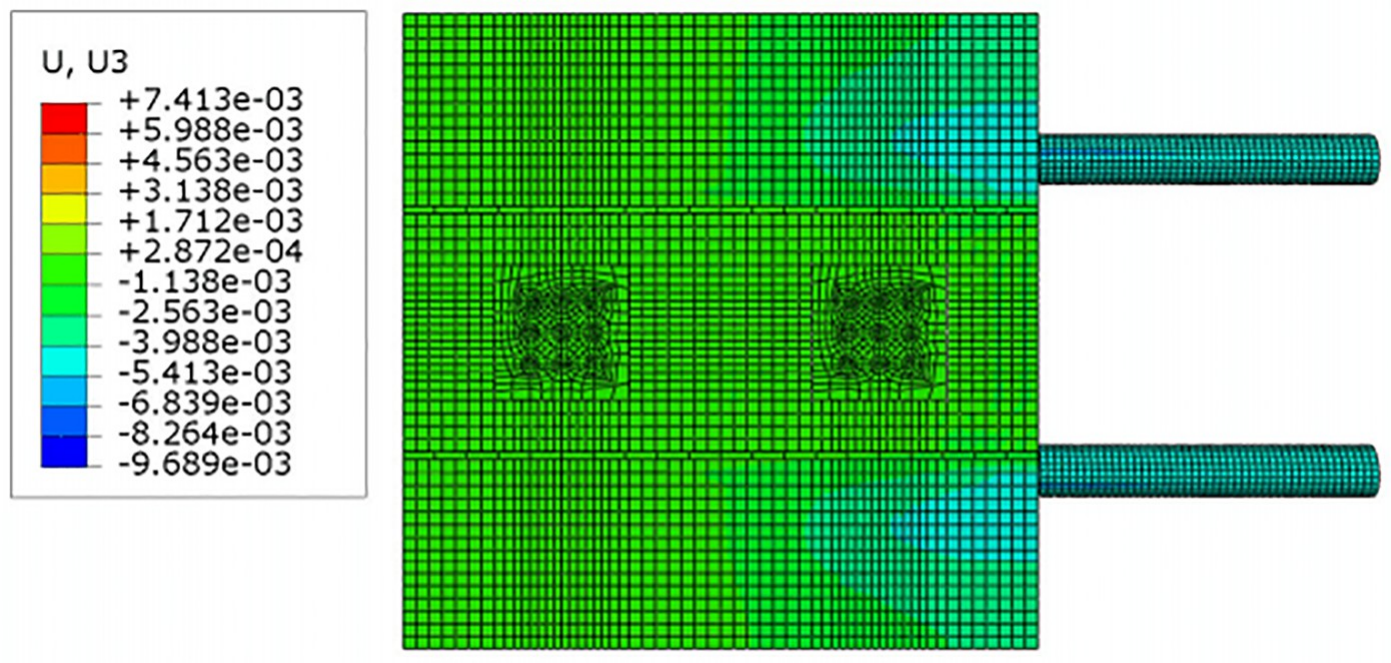
Model surface deformation clouds of loess-like soils at pipe jacking up to 5m.

**Fig 11:**
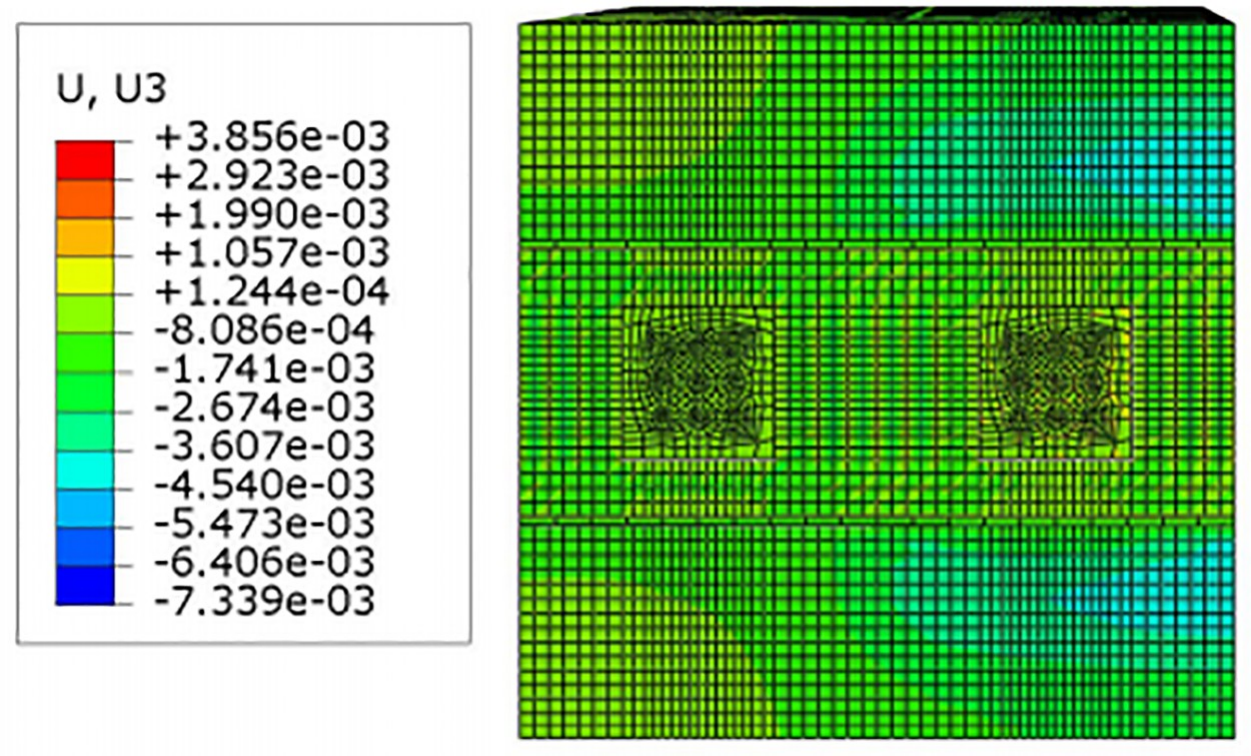
Model surface deformation clouds of loess-like soils at pipe jacking up to 10m.

**Fig 12:**
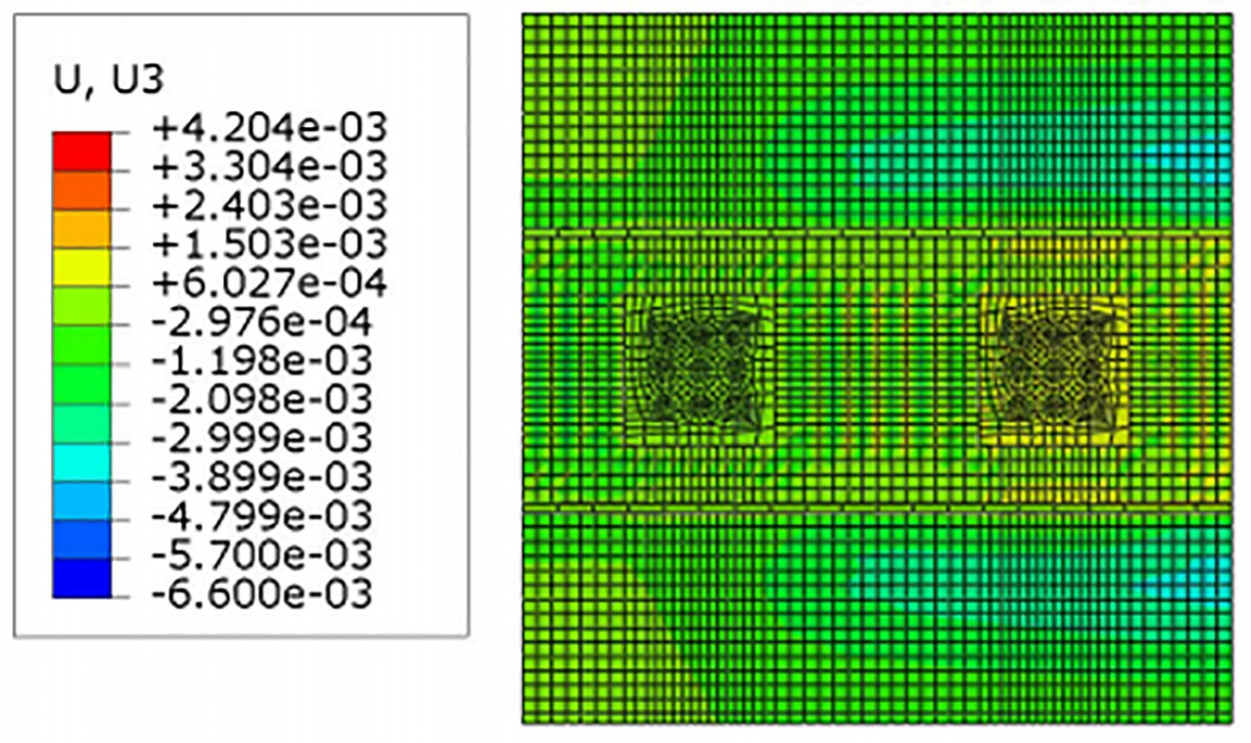
Model surface deformation clouds of loess-like soils at pipe jacking up to 20m.

**Fig 13:**
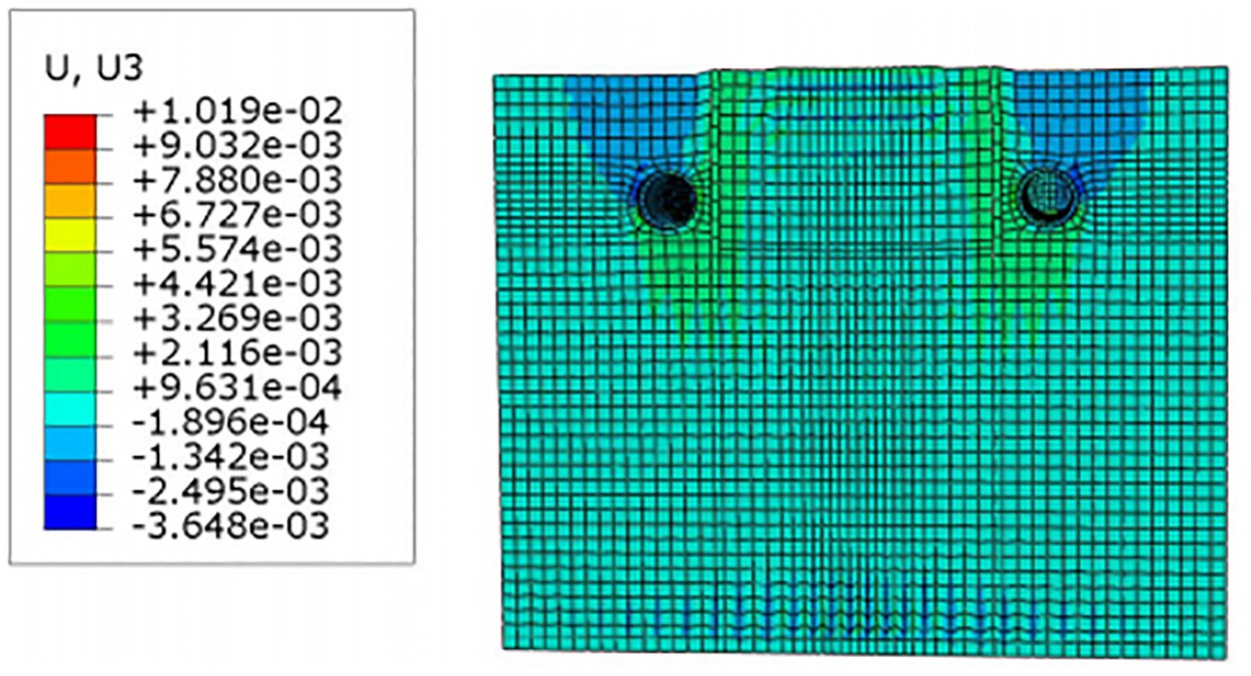
Model deformation of loess-like soils in the x-direction.

**Fig 14:**
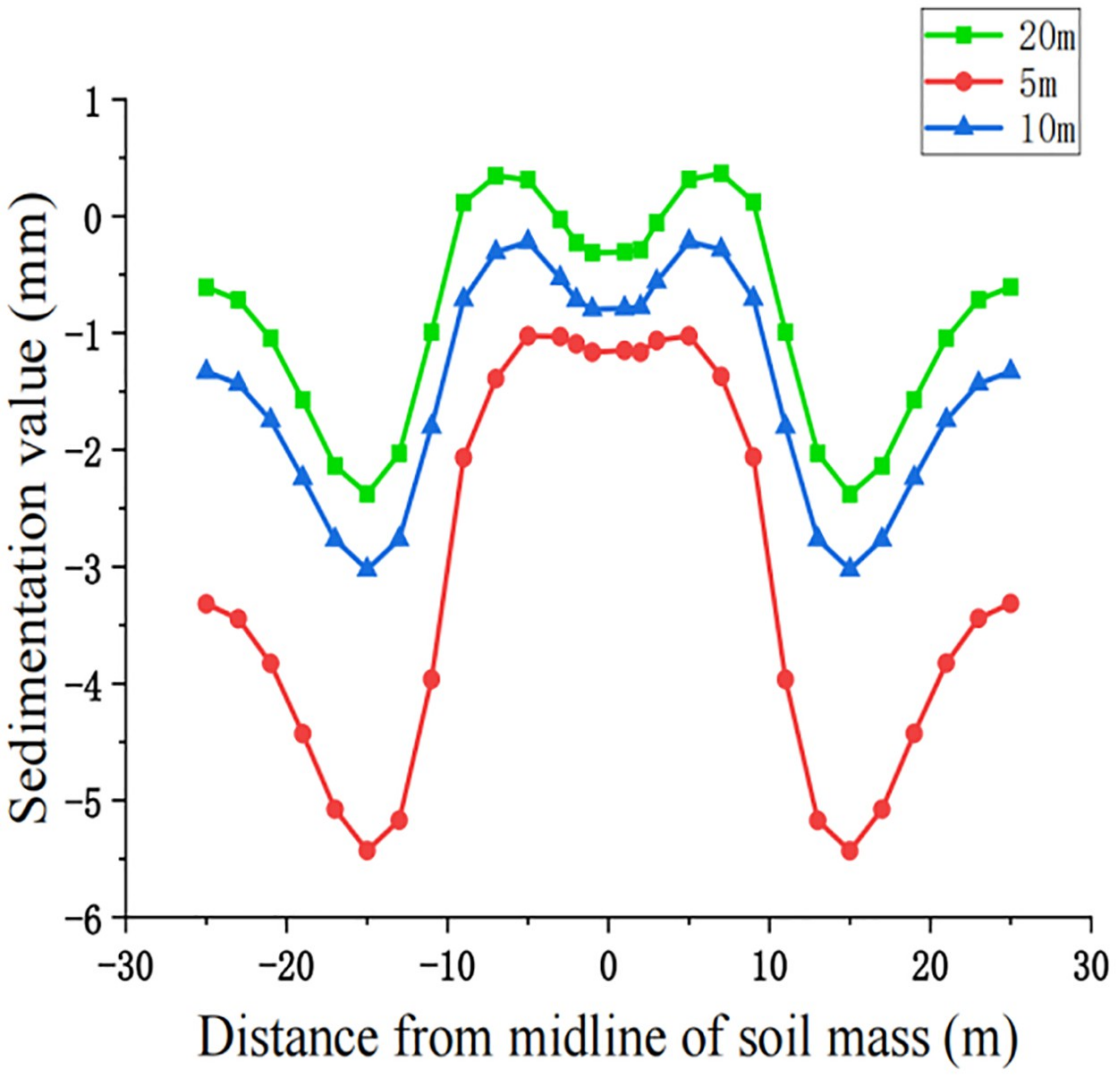
Surface deformation curves of loess-like soil under three working conditions.

**Fig 15:**
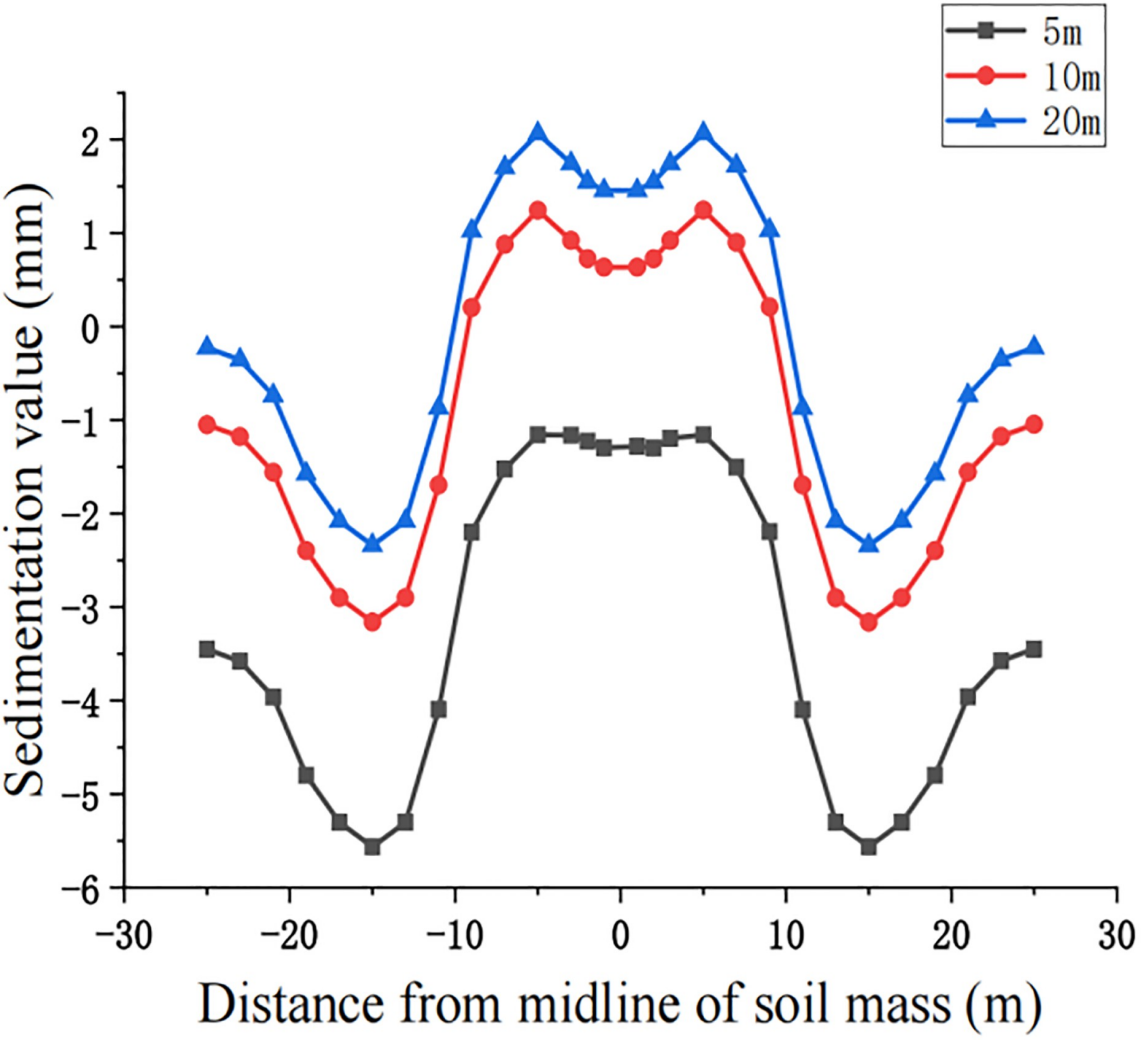
Surface deformation curves for three working conditions of clay.

**Fig 16:**
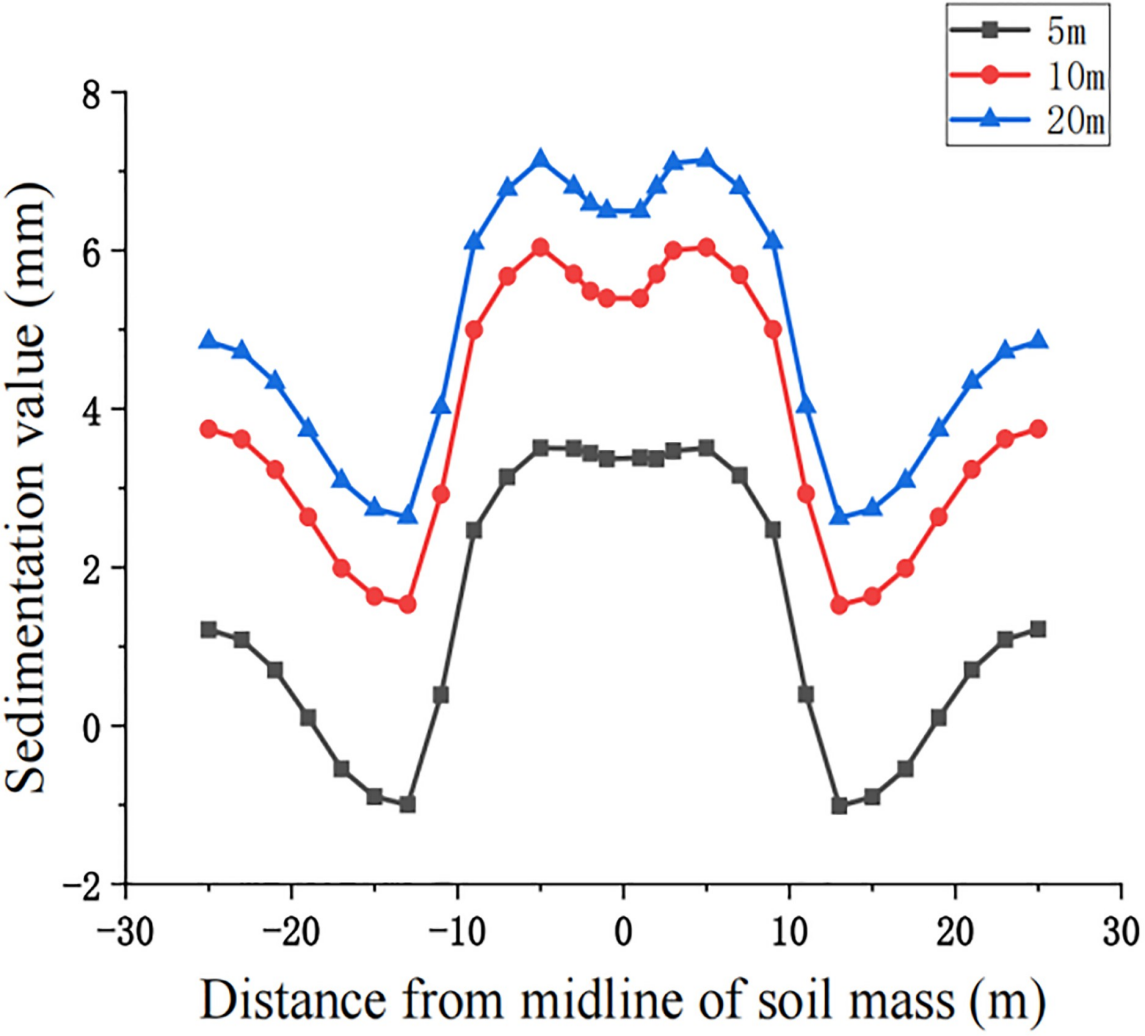
Surface deformation curves for three working conditions of powder soil.

**Fig 17:**
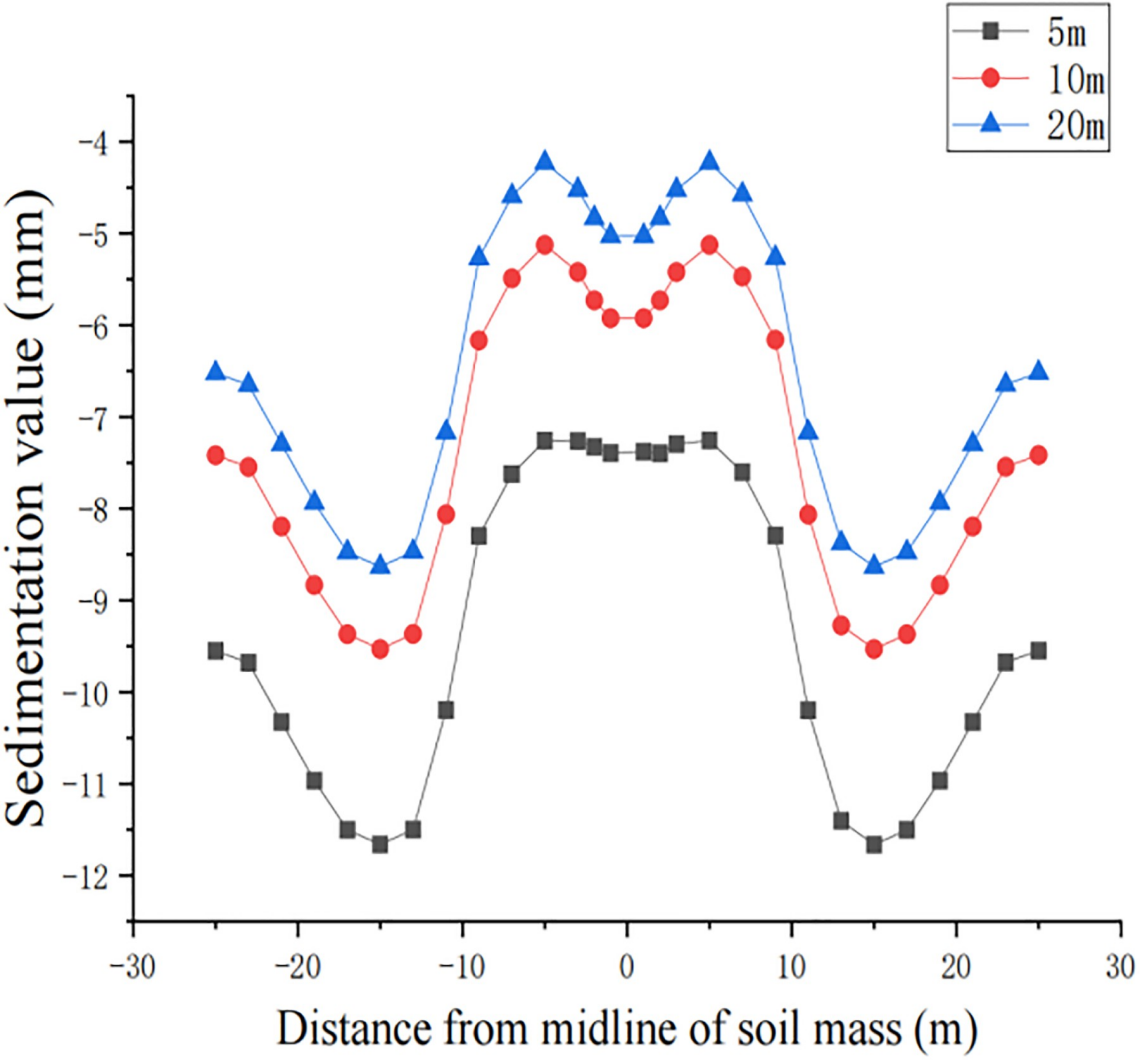
Surface deformation curves for three working conditions of pulverized clay.

For construction simulation, four groups of soil conditions were used, namely, loess-like soil, clay, powder soil and powder clay, and the soil part began to deform after jacking of the left and right line jacking pipes. The soil deformation models for the three working conditions are shown in Figs 10–13. The surface deformation curves of four soils under three working conditions are shown in Figs 14–17.

According to Fig 10 shows that when the pipeline is only pushed in for 5 m, the deformation only occurs near the tunnel entrance, and the soil near the bridge pier has no obvious force deformation. The cloud diagram Fig 11 analysis shows that as the jacking distance increases, the surface deformation range gradually deforms to the depths and is a settlement deformation, and the settlement of the tunnel entrance becomes increasingly obvious. A small settlement deformation of the soil can also be observed near the bridge pier near the isolation pile. Analysis of the surface deformation cloud Fig 12 shows that when it rises to 20 m, most of the ground surface has subsided along the pipeline direction, and the soil near the bridge piers has obvious settlement and small uplift deformation. Among them, the uplift of the soil near the piles is isolated. The analysis of Fig 13 shows that with the increase in the jacking distance, the settlement of the soil at the same section of the surface gradually decreases, and there is even a gradual upward trend near the isolation pile.

According to the analysis of the surface deformation curve in Figs 14–17, the settlement curves of the four types of soil are similar to two normal distribution curves, and the maximum settlement position is above the two pipelines and gradually decreases laterally to both sides along the pipeline. Due to the existence of the isolation pile, the soil settlement decreases when approaching the isolation pile. Compared with 10 m, the maximum settlement value at the selected node gradually decreases, the minimum settlement value of the soil near the bridge pier also decreases, and the middle soil settlement gradually appears on both sides above the center. The maximum settlement value of the surface node continues to decrease at 20 m compared with 10 m, but the decrease is also relatively smaller than that at 5 m~10 m. The law of surface deformation can be obtained preliminarily. At the same surface section position, as the jacking distance increases, the maximum settlement value above the two pipes gradually decreases, and the minimum settlement value of the soil near the bridge pier gradually decreases until there is slight uplift, while the central soil gradually settles.

**Fig 18:**
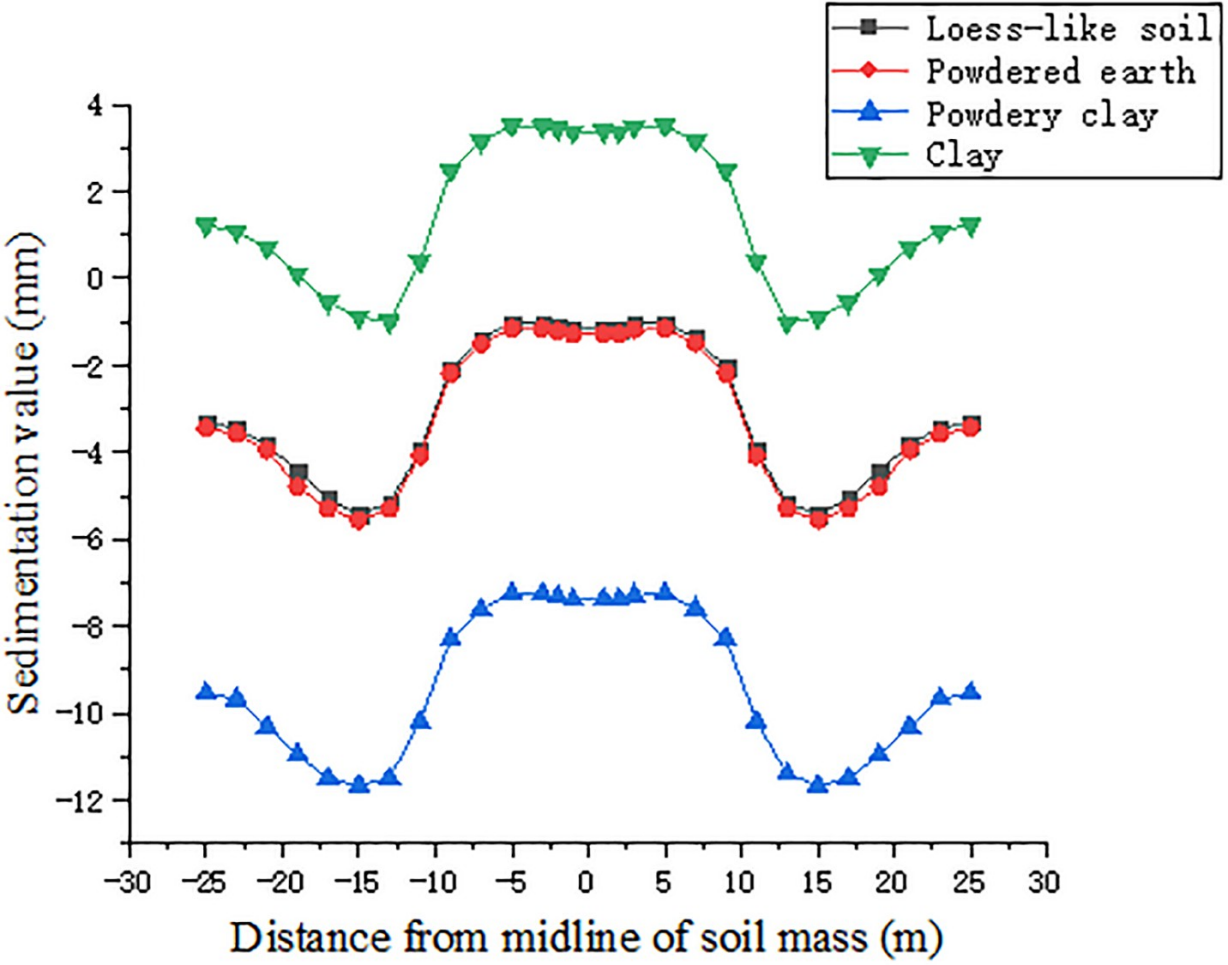
Longitudinal deformation curves of four soil surfaces jacked up to 5m.

**Fig 19:**
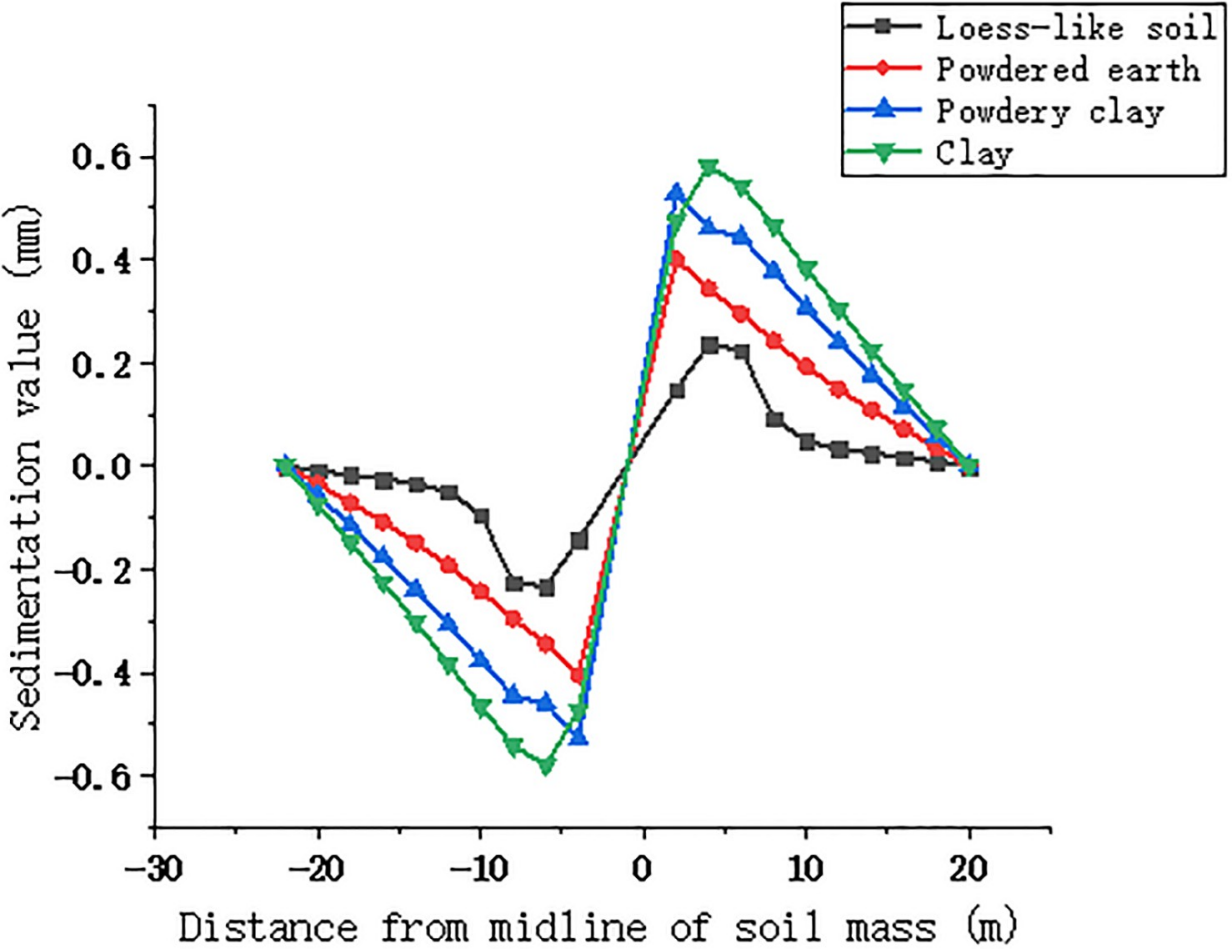
Transverse deformation curves of four soil surfaces jacked up to 5m.

The surface longitudinal and transverse deformation curves of the four soils selected for maximum settlement, i.e., topping out to 5 m, were compared, as shown in Figs 18 and 19.

According to the analysis in Fig 18, when the pipe jacking starts, the settlement of powder clay is the largest among the four soils, with the maximum value reaching 11.66 mm, while the settlement of powder soil is the smallest, only 1 mm, but its uplifted part is greater, and the maximum uplifted position is outside the isolation pile, with the uplifted value reaching 3.5 mm. Since the elastic modulus of powder clay is the smallest, its settlement is more concentrated compared with the other three soils, i.e., the soil above the pipeline is more dispersed, while the maximum settlement of powder soil is more concentrated and closer to the isolation pile than the other three soils. The location of the maximum settlement, i.e., the soil above the pipeline, is more dispersed, while the maximum settlement point of powdered clay is more concentrated and closer to the isolation pile than the other three soils. The settlement curves of loess-like soil and clay-like soil are more consistent, and the overall settlement of clay-like soil is approximately 0.3 mm greater than that of loess-like soil. Analyzing the transverse deformation curve Fig 19, it can be seen that the soil with the largest longitudinal deformation is powder soil, followed by powder clay, and loess-like soil has the smallest deformation. The maximum value of deformation of powder soil is 0.58 mm, and the maximum value of deformation of loess-like soil is 0.23 mm, with a difference of 0.35 mm. The comprehensive longitudinal and transverse deformation curves of the four soils can be summarized as follows: during the initial pipe jacking, the larger uplift value of powder soil, larger settlement of powder clay, and larger cohesion of clay compared with those of loess-like soil are not convenient for construction, so we obtain that loess-like soil is more suitable for single-chamber double-line large diameter pipe jacking construction.

### Simulation results of a single variable among the soil parameters

**Fig 20:**
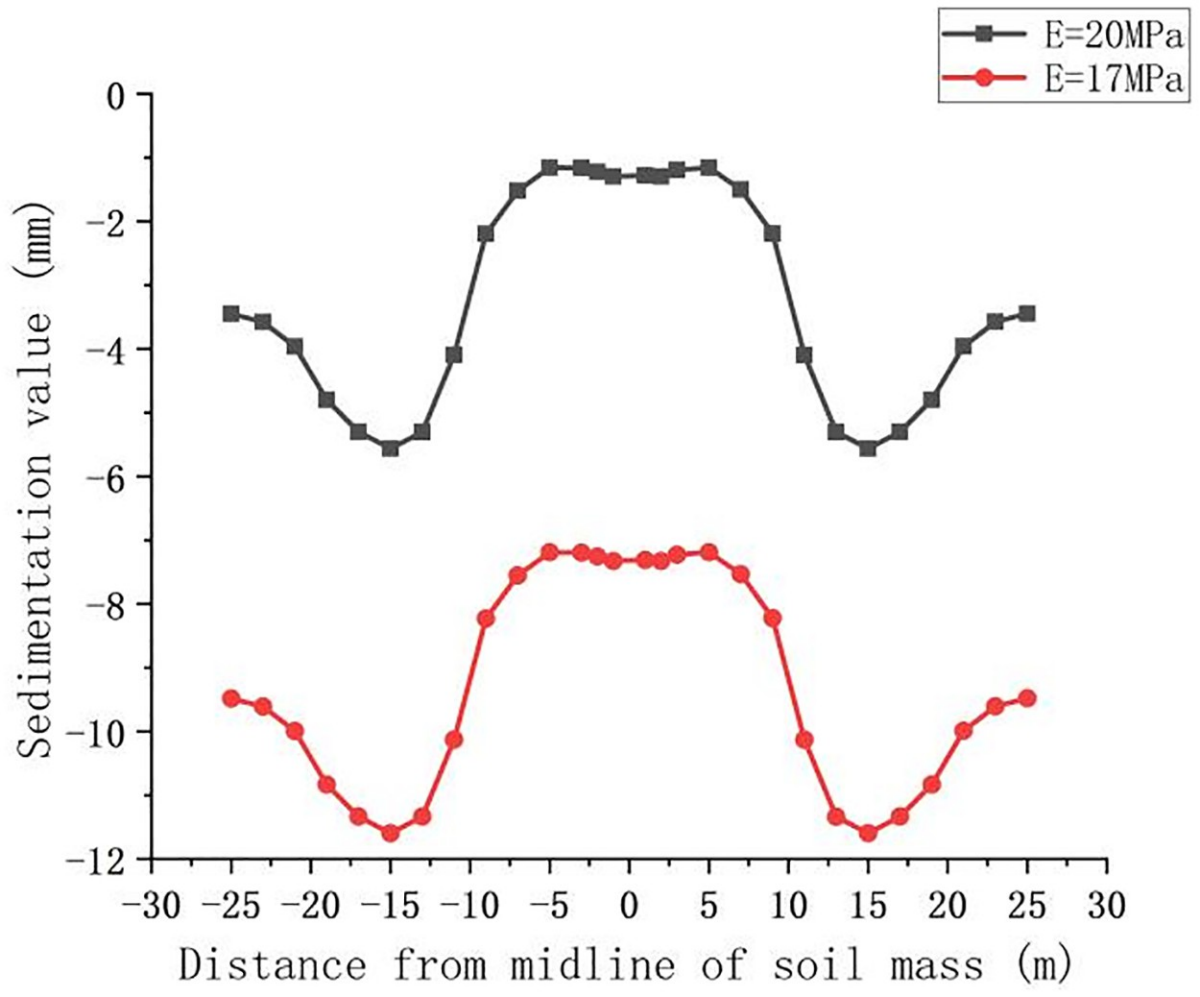
Deformation curve caused by the change in the elastic modulus.

**Fig 21:**
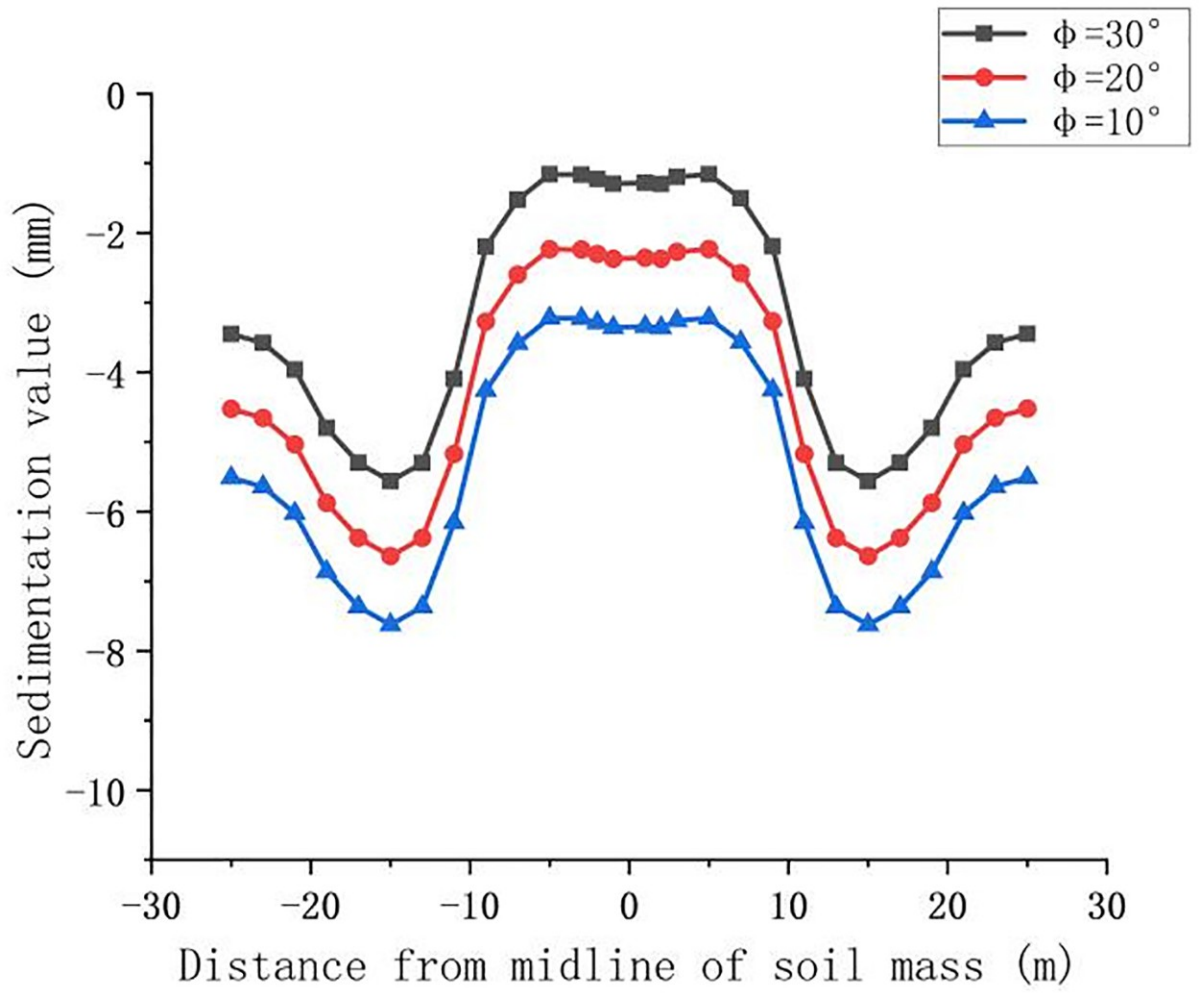
Deformation curve caused by the change in the internal friction angle.

This paper focuses on the influence of the elastic modulus and internal friction angle on the ground settlement. On the basis of the original model, the loess-like soil crossed by the actual construction is selected as the basic parameter. To simplify the calculation of the influence of significant variables, the overlying soil is simplified into one layer. When the simulation is set to 10 m, the other parameters of the soil are not changed and the corresponding parameters are changed to obtain the law of settlement influence. The elastic moduli are 17 MPa and 20 MPa, and the internal friction coefficients are 10°, 20°, and 30° for the simulation of land subsidence. The Z-direction settlement value of the surface section after the pipe jacking passes through is analyzed. The results of the surface deformation curve caused by the change in the elastic modulus and the change in the internal friction angle are shown in Figs 20 and 21.

Analysis of the settlement curve Fig 20 shows that the maximum value of settlement changes from 5.9 mm to 11.7 mm with only a 3 MPa change in the modulus of elasticity, which is approximately 5 mm. Therefore, in the actual construction of the soil layer survey, the soil layer with a larger modulus of elasticity and reasonable jacking in the head of the pipe jacking machine is selected. Analysis of the settlement curve Fig 21 shows that jacking to 10 m in the case of a constant modulus of elasticity E changes the angle of internal friction φ. The angle of internal friction on the surface of the settlement changes in the range of 10–30°, which is not large. The greater the angle of internal friction is, the smaller the upper surface deformation, and vice versa. The smaller the angle of internal friction is, the greater the value of settlement deformation, but the increase or decrease in value is not large. When the difference in the angle of internal friction is 10°, the settlement deformation value change is approximately 1.7 mm. Because the change value is small, when the actual construction attempts to reduce the amount of settlement by changing the angle of internal friction with the soil, it is necessary to compare the economic efficiency.

### Effect of pipe jacking depth on surface settlement

**Fig 22:**
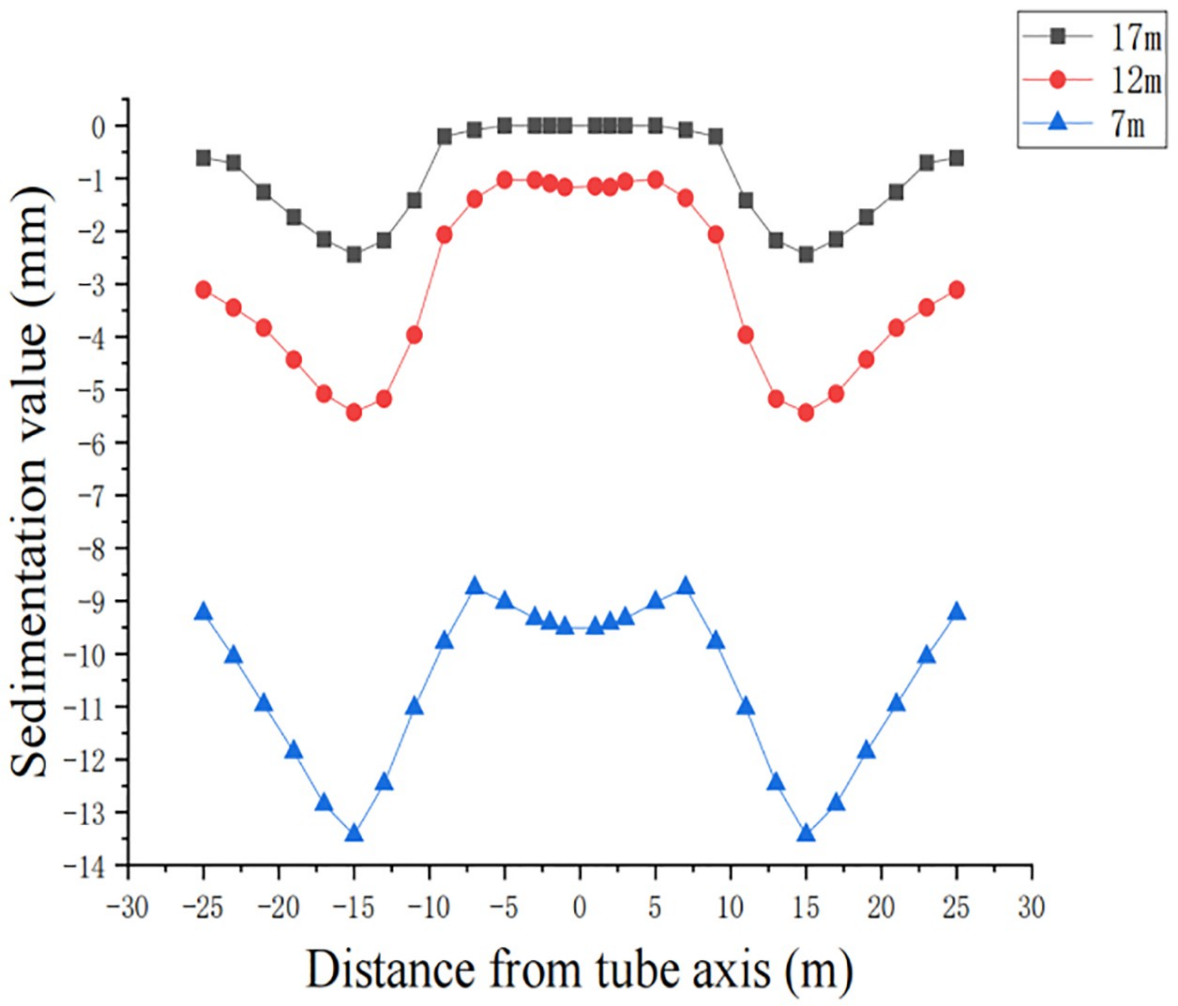
Surface settlement curves for different pipe jacking depths.

From the above analysis, it can be seen that single-chamber double-line pipe jacking is more suitable for loess-like construction, so loess-like parameters are selected to analyze the impact of double-line pipes with different burial depths on engineering construction. Taking h = 7 m, h = 12 m, and h = 17 m, the other parameters are kept the same as those of loess-like soil. The calculation result is shown in Fig 22.

According to the analysis in Fig 12, the burial depth has a greater impact on surface settlement. As the burial depth of the pipeline increases, the lateral influence range of surface settlement gradually increases, but the amount of settlement gradually decreases. When the burial depth is large, there is almost no settlement of the soil above the axis of the two pipelines, and the overall settlement is also small. When the burial depth is small, the soil mass and the overall settlement of the two pipeline axes are larger. The mechanism of the effect of burial depth on surface settlement in actual construction is as follows: the thinner the overburden is, the lower the earth pressure, the more sensitive the overburden is to disturbances such as thrust, frictional resistance, and stress release, and the easier it is to deform. According to research, for the ultra shallow layer, the damage to the ground is greater, but the scope of influence is small. Therefore, when designing the pipe jacking pipeline, it should be fully integrated and designed with a reasonable burial depth. In addition, when the burial depth is too small, such as 7 m in the figure, the soil above the axis of the two pipelines will have a large settlement of approximately 10 mm. When single-chamber double-line large diameter pipe jacking is used for construction, the pipe jacking radius is 3–4 m. According to the simulation analysis, considering the workload and the impact on the surrounding soil, the burial depth is generally set to be greater than or equal to twice the diameter of the pipe, that is, the burial depth of the pipe is greater than 8 m.

## Conclusion

1. The comparison of numerical simulation, finite element simulation and field monitoring proved that the finite element simulation is closer to the actual construction. The settlement curve of the surface soil caused by the construction of large diameter pipe jacking with single-lumen and double-line construction approximates two normal distributions, its maximum settlement value appears above the two pipelines, and the minimum settlement value appears near the isolation piles around the building, which can prove that the existence of isolation piles greatly reduces the impact of pipe jacking construction on the building. The difference between the maximum and minimum values of surface deformation of the four soils is approximately 4 mm, and the settlement value above the pipe accounts for approximately 45% of the total settlement, so the key to controlling the settlement is the soil above the pipe.
2. By comparing the deformation curves of the four soils, we find that for the longitudinal deformation, the settlement value of powdery clay is the largest and the maximum settlement point is more dispersed, up to 11.66 mm, while the settlement value of powdery clay is the smallest and the settlement point is more concentrated, only 1 mm, but its elevated part is greater, i.e., the elevated value is 3.5 mm. The settlement curves of loess-like soil and clay-like soil are basically the same, and the overall settlement of the latter is approximately 0.3 mm greater than that of the former. For lateral deformation, the soil with the largest deformation is powdery clay, followed by clay, and loess has the smallest deformation. The maximum value of deformation of powdery clay is 0.58 mm, and the maximum value of deformation of loess-like soil is 0.23 mm, with a difference of 0.35 mm. According to the above analysis, it is known that the uplift value of powdery clay is larger, the settlement of powdery clay is larger, and the adhesion of clay is larger than that of loess. Therefore, loess-like soils are more suitable for single-chamber double-line large diameter pipe jacking construction.
3. It can be known by analyzing the changes in individual soil parameters that when the angle of internal friction varies in the range of 10–30°, the larger its value is, the smaller the soil deformation, but the variation is only 1.7 mm, so the variation in the angle of internal friction has little effect on the surface soil deformation. The larger the elastic modulus of the soil is, the smaller the amount of ground settlement caused, and conversely, the smaller the elastic modulus of the soil is, the larger the ground settlement caused. When changing the burial depth, it is found that the greater the burial depth of the top pipe is, the smaller the settlement of the surface soil, but the greater the lateral influence on the soil deformation, and vice versa. When the burial depth of pipe jacking is too small, the soil above the axis of the two pipes will produce a large settlement. Therefore, in single-chamber double-line large diameter pipe jacking construction, when the pipe diameter is 3–4 m, the burial depth of the pipe should be greater than 8 m.

## Supporting information

S1 Fig Schematic diagram of soil loss location. Schematic diagram of soil loss during jacking of pipe jacking shown according to Peck’s formula.
(PNG)

S2 Fig Cross section of soil in X direction. Schematic diagram of the cross section of the established soil model in the x-direction.
(PNG)
