# Supplementary figures and images for: Simulation analysis of the effect of single-chamber double-line pipe jacking through different soil materials on surface uplift and subsidence
Source: PLoS One. 2022 Oct 21;17(10):e0276366. doi: 10.1371/journal.pone.0276366 (PMC9586396; doi:10.1371/journal.pone.0276366)

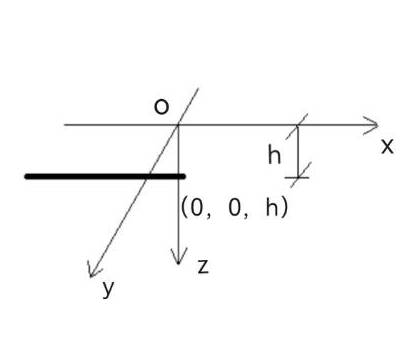

Supplement: S1 Fig — Schematic diagram of soil loss during jacking of pipe jacking shown according to Peck’s formula. (PNG) [file pone.0276366.s001.png]

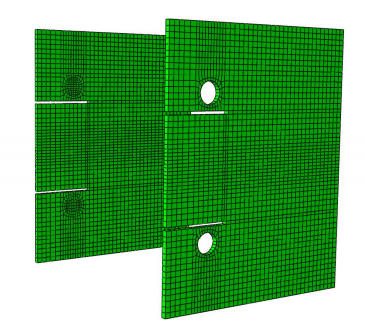

Supplement: S2 Fig — Schematic diagram of the cross section of the established soil model in the x-direction. (PNG) [file pone.0276366.s002.png]
